# Vangl-dependent Wnt/planar cell polarity signaling mediates collective breast carcinoma motility and distant metastasis

**DOI:** 10.1186/s13058-023-01651-2

**Published:** 2023-05-05

**Authors:** Kacey VanderVorst, Courtney A. Dreyer, Jason Hatakeyama, George R. R. Bell, Julie A. Learn, Anastasia L. Berg, Maria Hernandez, Hyun Lee, Sean R. Collins, Kermit L. Carraway

**Affiliations:** 1 Department of Biochemistry and Molecular Medicine and University of California Davis Comprehensive Cancer Center, University of California Davis School of Medicine, Sacramento, CA USA; 2 Institute for Stem Cell Biology and Regenerative Medicine, Stanford University School of Medicine, Stanford, CA USA; 3 Department of Microbiology and Molecular Genetics, University of California Davis, Davis, CA USA

**Keywords:** Collective cell migration, Planar cell polarity, Non-canonical Wnt, Metastasis

## Abstract

**Background:**

In light of the growing appreciation for the role of collective cell motility in metastasis, a deeper understanding of the underlying signaling pathways will be critical to translating these observations to the treatment of advanced cancers. Here, we examine the contribution of Wnt/planar cell polarity (Wnt/PCP), one of the non-canonical Wnt signaling pathways and defined by the involvement of the tetraspanin-like proteins Vangl1 and Vangl2, to breast tumor cell motility, collective cell invasiveness and mammary tumor metastasis.

**Methods:**

Vangl1 and Vangl2 knockdown and overexpression and Wnt5a stimulation were employed to manipulate Wnt/PCP signaling in a battery of breast cancer cell lines representing all breast cancer subtypes, and in tumor organoids from MMTV-PyMT mice. Cell migration was assessed by scratch and organoid invasion assays, Vangl protein subcellular localization was assessed by confocal fluorescence microscopy, and RhoA activation was assessed in real time by fluorescence imaging with an advanced FRET biosensor. The impact of Wnt/PCP suppression on mammary tumor growth and metastasis was assessed by determining the effect of conditional Vangl2 knockout on the MMTV-NDL mouse mammary tumor model.

**Results:**

We observed that Vangl2 knockdown suppresses the motility of all breast cancer cell lines examined, and overexpression drives the invasiveness of collectively migrating MMTV-PyMT organoids. *Vangl2*-dependent RhoA activity is localized in real time to a subpopulation of motile leader cells displaying a hyper-protrusive leading edge, Vangl protein is localized to leader cell protrusions within leader cells, and actin cytoskeletal regulator RhoA is preferentially activated in the leader cells of a migrating collective. Mammary gland-specific knockout of *Vangl2* results in a striking decrease in lung metastases in MMTV-NDL mice, but does not impact primary tumor growth characteristics.

**Conclusions:**

We conclude that Vangl-dependent Wnt/PCP signaling promotes breast cancer collective cell migration independent of breast tumor subtype and facilitates distant metastasis in a genetically engineered mouse model of breast cancer. Our observations are consistent with a model whereby Vangl proteins localized at the leading edge of leader cells in a migrating collective act through RhoA to mediate the cytoskeletal rearrangements required for pro-migratory protrusion formation.

**Supplementary Information:**

The online version contains supplementary material available at 10.1186/s13058-023-01651-2.

## Background

Distant metastasis is a complex, multi-step process whereby cancer cells invade surrounding tissues, access and traverse the vasculature, disseminate throughout the body, and proliferate at secondary sites [1]. Observations that carcinoma cells invade almost exclusively in a collective manner [2], and that metastatic lesions may be largely seeded by polyclonal cell clusters rather than individual disseminated cells [3–6], strongly suggest that collective cell migration, defined as the coordinated movement of cohorts of cells in sheets or clusters that retain cell–cell contacts [7], is a major driver of invasiveness and metastasis. In non-transformed tissues, collective cell migration promotes blood vessel formation [8], convergent extension [9], branching morphogenesis [10], and wound healing [7]. However, the study of collective cell migration in carcinomas significantly lags that of classical epithelial–mesenchymal transition (EMT)-mediated motility of individual cells. A better understanding of cell signaling pathways that govern collective cell migration and invasiveness may identify novel therapeutic targets for intervention in patients with aggressive and late-stage disease.

We recently proposed a model whereby aberrant activation of Wnt/planar cell polarity (Wnt/PCP) signaling [11], a branch of non-canonical Wnt signaling paradoxically critical to both the establishment and maintenance of polarity in static epithelial sheets as well as cell migration during embryonic development [12, 13], promotes the invasiveness of primary tumor cells. In Wnt/PCP signaling, binding of non-canonical Wnt ligands such as Wnt5a to transmembrane Frizzled (Fzd) receptors initiates polarization signals that are transduced through the essential pathway components Vangl, Dishevelled (Dvl), and Prickle (Pk) [14–16]. Although Dvl and Fzd are required for both canonical and alternative non-canonical Wnt pathways, Vangl1 and Vangl2 transmembrane scaffolds likely provide the platform necessary for the assembly of pathway-specific complexes [17] and thus distinguish Wnt/PCP from other Wnt signaling pathways. Vangl1 and Vangl2 are highly similar; their amino acid sequences exhibit 64.3% identity and 78.6% similarity, and no functional biochemical differences have been reported [18]. However, Vangl2 alterations result in more profound developmental defects, suggesting a more prominent role for Vangl2 in embryonic tissue organization [18, 19]. Wnt/PCP signaling is a significant driver of collective cell migration during development [20, 21], and studies employing Looptail (Lp) mice, which harbor point mutations in Vangl2 that alter its trafficking and localization, suggest that Vangl subcellular localization is critical in collectively migrating cells [22].

Consistent with observations from developmental studies, Wnt/PCP components mediate cell motility in cancer cells [23, 24], and core Wnt/PCP components are dysregulated in multiple tumor types, including breast [25–31], brain [32], ovarian [24], prostate [33], gastric [23], and colorectal cancers [34, 35]. We have reported that *VANGL1* and *VANGL2* are, respectively, upregulated in 5% and 24% of invasive breast carcinomas compared to healthy breast tissue, [18] and others have found that elevated *VANGL1* and *VANGL2* are also associated with increased recurrence and decreased metastasis-free survival of breast cancer patients [28, 31]. Together, these observations point to the possibility that aberrant Wnt/PCP activation contributes to breast cancer progression by promoting collective cell migration resulting in metastasis.

Here we examine the role of Vangl-dependent Wnt/PCP signaling in breast cancer invasiveness and metastasis. We demonstrate that Vangl2 is critical for efficient metastasis but dispensable for primary tumor growth in ErbB2-induced mouse mammary tumors. We further find that Vangl-dependent Wnt/PCP signaling at the leading edge of migrating breast cancer cells results in increased RhoA GTPase activity and formation of pro-migratory protrusions, resulting in collective cell migration in vitro and invasion ex vivo.

## Methods

### Generation of Vangl2/NDL mice

All experimental protocols were approved by the IACUC of the University of California, Davis, CA, USA. *Vangl2*^*tm*^^2^^*.1Mdea*^*/J* conditional knockout mice [36] (The Jackson Lab, Stock #025174) were crossed with *Tg(MMTV-cre)4Mam/J* mice [37] (The Jackson Lab, Stock #003553) to generate mice with Vangl2 deletion in the mammary gland. The *MMTV-NDL* mouse has been previously described [38]. Genotypes were confirmed by polymerase chain reaction in house using primers for Vangl2 (Fwd 5’-CAGAA CCTCCTGTCCCTGA-3’; Rev 5’-CTCAGCTAAACCACCTCTGC-3’), Cre (Fwd 5’-GCGGTCTGGCAGTAAAAACTATC-3’; Rev 5’-GTGAAACAGCATTGCTGTCACTT-3’), and NDL (Fwd 5’-TTCCGGAACCCACATCAG -3’; Rev 5’- GTTTCCTGCAGCAGCCTA -3’).

### Tumor monitoring and analysis

Mammary tumors were palpated once or twice weekly in female Vangl2/NDL mice commencing at 16 weeks of age by a single investigator and all palpable tumors were measured by calipers. When the largest tumor reached 2 cm in any direction, mice were euthanized by CO_2_ asphyxiation and tumors were collected and either fixed in 10% neutral buffered formalin for paraffin embedding and sectioning or further dissociated for in vitro analysis. Mice with illnesses arising independent of their tumors that required killing prior to reaching the pre-determined endpoint were excluded from analyses.

### Histology and immunohistochemistry

Histologic analysis of lungs was performed for all mice in the study (n = 20 per genotype) and for a randomly selected subset of Vangl2^+/+^/NDL and Vangl2^fl/fl^/NDL mammary tumors (n = 4 per genotype). H&E-stained sections were prepared using previously described methods [39]. Immunohistochemistry (IHC) was performed as previously described [40]. An internal negative control (no primary antibody) was included with each assay.

### Lung metastasis analysis

Lungs were inflated with PBS, fixed in 10% neutral buffered formalin, paraffin-embedded, and sectioned for IHC analysis and H&E staining. The number of ErbB2-positive metastatic lesions present on all five lung lobes was counted for all mice in the study (n = 20 per genotype) from images taken on a Keyence BZ-X810 microscope. Metastatic burden was quantified by normalizing the number of metastatic lesions to the total tumor burden.

### Tail vein injections

Pooled primary Vangl2^+/+^/NDL and Vangl2^fl/fl^/NDL mammary tumors (Vangl2^+/+^/NDL* n* = 11, Vangl2^fl/fl^/NDL *n* = 10; 5 × 10^5^ in 200 µL PBS) were instilled to the lateral tail vein of 12-week-old FvB/NJ mice. Lungs were harvested 6 weeks post-injection and analyzed for metastatic lesions. Mice were randomly assigned to cohorts and were caged as mixed cohorts.

### Cell culture and reagents

BT549, MDA-MB-231, MDA-MB-468, SkBr3, MCF7, T47D, nMuMG, L-Cells, L-Cells-Wnt3a, and HEK293T cells were purchased from the American Type Culture Collection (ATCC) and maintained as recommended at 37 °C in 10% CO_2_ in media supplemented with 10% fetal bovine serum (FBS, Genesee Scientific), 1% penicillin–streptomycin (Invitrogen). Met-1 (gifted by A.D. Borowsky) and NDL cells were maintained as previously described [41, 42]. Prior to use, cell lines were authenticated by short-tandem repeat profiling (Genetics Core Facility; University of Arizona, Tucson, AZ, USA) and tested for mycoplasma contamination by RT-PCR as described [43, 44]. Antibodies used for immunoblotting, immunofluorescence, and immunohistochemistry are as follows: anti-Dvl2, anti-p-β-Catenin (Ser33/37/Thr41), anti-β-Catenin (Cell Signaling), anti-Tubulin (Sigma), anti-Vangl2 (Proteintech) and anti-p-Vangl2 (Ser79/82/84) (Invitrogen) anti-Actin (Sigma), horseradish peroxidase-conjugated goat anti-mouse and goat anti-rabbit secondary antibodies (Bio-Rad), anti-Vangl1 (R&D Systems), anti-Fzd7 (Abcam), anti-Keratin14 (Biolegend), anti-E-Cadherin, anti-Flag and anti-V5 (Cell Signaling), anti-Phalloidin647 (Invitrogen), and AlexaFluor 488-conjugated goat anti-mouse, AlexaFluor 546-conjugated goat anti-rabbit, and Alexa-Fluor 568-conjugated goat anti-chicken secondary antibodies (Invitrogen), and anti-Ki67, anti-c-Caspase3, and anti-ErbB2 (Cell Signaling). Cells were treated with the Wnt-inhibitor C59 at 100 nM (R&D Systems).

### Generation of stable overexpression and knockdown cell lines by lentiviral transduction

*VANGL2*-targeted shRNA constructs shVangl2-1 (human, ID: V3LHS_334647 or ID: TRCN0000180101, mouse, ID: TRCN0000124569), shVangl2-2 (human, ID: V3LHS_334648 or TRCN0000417141, mouse, ID: TRCN0000124572) (Dharmacon, Sigma-Aldrich), or Scrambled control vectors pGIPZ (Dharmacon) or pLKO.1 (a gift from David Sabatini, Addgene plasmid #1864; http://n2t.net/addgene:1864; RRID:Addgene_1864) were employed for Vangl2-depletion studies. Stable overexpression cells were created using Vangl1 and Vangl2 plasmids (Harvard PlasmID repository, HsCD00339551 and HsCD00294893) and Wnt5a plasmid that was a gift from Marian Waterman (Addgene plasmid # 35,911; http://n2t.net/addgene:35911; RRID:Addgene_35911) subcloned into the control vector pLX304, a gift from David Root (Addgene plasmid # 25,890; http://n2t.net/addgene:25890; RRID:Addgene_25890). VSVG-pseudotyped lentivirus was generated by transfecting HEK293T cells with the psPax2 packaging vector. Cells were transduced with 10 µg/mL polybrene (Millipore), followed by drug selection with 1 µg/mL Puromycin (Sigma-Aldrich) or 4 µg/mL Blasticidin (Sigma-Aldrich).

### Wnt5a and Wnt3a stimulation

Wnt5a-conditioned media was produced by stably transducing nMUMG, MCF7, MDA-MB-231, MDA-MB-468, SkBr3, or NDL cells with Vector- or Wnt5a-containing lentivirus. Vector- or Wnt3a-conditioned media was collected from L-Cell and L-Cell-Wnt3a, respectively. Conditioned media was collected from confluent cell culture plates, cleared of debris by centrifugation, and stored at − 80 °C.

### Scratch migration assays

Confluent monolayers of cells were scratched with a sterile pipette tip and imaged immediately and after 12 h with an Olympus IX81 microscope with cellSens Entry software. The scratch area was measured using ImageJ (NIH), and the area of the scratch filled in over 12 h was quantified. Results were normalized to appropriate controls for each assay.

### Immunoblotting

Cells were washed with 1X PBS and lysed directly in 2 × Laemmli sample buffer. All samples were resolved by SDS-PAGE, transferred to nitrocellulose membranes, and blotted with the indicated antibodies. Immunoblots were developed using Pierce SuperSignal West chemicals (Thermo Fisher) on an Alpha Innotech imaging station and quantified with ImageJ (NIH).

### Real-time PCR analysis

RNA was collected using a PureLink RNA MiniKit (Ambion) and converted to cDNA with the High-Capacity cDNA reverse transcription kit (Applied Biosystems). *q*PCR was conducted in a Bio-Rad CFX96 real-time PCR system using TaqMan gene-specific primer/probe sets (Applied Biosystems) and SsoAdvanced master mix (Bio-Rad). Analysis was conducted using Bio-Rad CFX Manager software, and message levels were normalized to GAPDH.

### Ex vivo 3D organoid invasion assays

*MMTV-PyMT* tumor samples were a kind gift from Dr. Jason Hatakeyama (Stanford University, Stanford, CA, USA). Tumors were dissociated into single cells as previously described [45] with minor modifications and seeded in Matrigel (Corning) with organoid growth media, which has been previously described [46]. After 24 h in Matrigel, cells were transduced with specified lentivirus and spinfected for 1 h at ~ 500G in a Beckman centrifuge. After seven days in culture, organoids derived from single *MMTV-PyMT* tumor cells were recovered from Matrigel using Cell Recovery Solution (Corning) and embedded into rat-tail collagen I (Thermo Fisher) as previously described [46]. Invasive protrusions were imaged and counted with an Olympus IX81 microscope with cellSens Entry software or Zeiss LSM 710 AxioObserver confocal microscope.

### RNAseq data mining

Raw RNAseq reads from Cheung et al., archived as SRP066316, were downloaded from the Sequence Read Archive [6]. Reference genome for pseudoalignment was built in Kallisto v0.43.1 from Genome Reference Consortium Mouse Build 38 using a k-mer length of 31. Reads were then pseudoaligned to the reference genome using 100 bootstraps to estimate error. Differential expression analysis was then performed in R using the DESeq2 package (1.28.1). Biological replicate #3 (SRR291722 and SRR2921727) varied considerably from the other replicates by principal component analysis and expression of key marker genes and was therefore omitted from the analysis.

### Immunofluorescence microscopy

Cells were seeded onto coverslips, fixed with 4% paraformaldehyde, and stained as indicated. PyMT-derived organoids embedded in collagen I were fixed with 4% PFA and stained as indicated. Imaging was conducted on a Zeiss LSM 710 AxioObserver confocal microscope or Keyence BZ-X810 microscope. An internal negative control (no primary antibody) was included with each assay. The average number of Vangl1-rich protrusions/cell was quantified by counting the number of Vangl1 + protrusions in leader cells along the leading edge of MCF7 cells actively migrating into a scratch made in a confluent monolayer from 4 or 8 independent scratch assays. The percentage of cells with Vangl1-rich protrusions was quantified by counting the number of leader cells with and without Vangl1 + protrusions along the leading edge of MCF7 cells actively migrating into a scratch made in a confluent monolayer from 4 or 8 independent scratch assays.

### FRET biosensor scratch assay imaging

Rac1 and RhoA intramolecular FRET biosensors have been previously described [47]. MCF7 cells stably expressing *VANGL2-*targeted shRNAs to deplete Vangl2 were stably transfected with Rac1 or RhoA intramolecular FRET biosensors using PEI transfection reagent. Rac1 or RhoA biosensor-expressing cells were sorted with a BD “inFlux” 18-color cell sorter (Becton Dicksinson). For all imaging experiments, cells were plated on glass-bottomed 96-well plates (Cellvis) and grown to confluency. Prior to imaging, the monolayers were scratched with a sterile pipette tip, and the media was replaced with Liebovitz-15 (L-15) media, which was made with no riboflavin, folic acid or dyes to reduce autofluorescence from the media supplemented with 2% FBS (UC Davis Biological Media Services). The plates were then transferred to a Nikon Eclipse TI equipped with an OKO Labs cage incubator set to 37 °C. The microscope is controlled by MATLAB (version 2015 A) through Micro-Manager (v 1.6), allowing precise, repeatable experiments. The X & Y stage positions of the scratch were identified by the user, and all scratch positions were stored in MATLAB for time-lapse imaging. Epifluorescent CFP/YFP FRET images were collected every 15 min for 12 h using a 20 × Nikon Apochromat 0.75 NA objective. Cyan (~ 440 nm) excitation illumination was provided by the X-Cite XLED1 BLX module, while the simultaneous acquisition of FRET images was achieved using dual Andor Zyla 4.2 sCMOS cameras separated by a Cairn TwinCam LS image splitter with a Chroma Technologies dichroic mirror (ZT491rdc) that reflects wavelengths less than 502 nm to one camera, while passing longer wavelengths to the second camera.

### Camera and illumination corrections

The dark-state noise for each camera was empirically measured as described [48]. In brief, several images were captured without illumination and the microscope light path set to the oculars. The dark-state correction image was generated by taking the median over the stack of dark images. This correction was then subtracted from all experimental images. CFP/YFP FRET ratio images were observed to have a gradient of activity from the top to the bottom of the images. A correction image was developed to remove this gradient as described [48]. Images of unstimulated, confluent monolayers of MCF7 cells expressing the CFP/YFP FRET sensor were collected. FRET ratio images were generated from raw CFP and YFP images that were processed using our standard analysis pipeline. The median FRET ratio was taken over the stack of images on a pixel-by-pixel basis. Only pixels that overlapped with a cell logical mask were included in the median analysis. To reduce local variability effects and noise, the median image was broken into 24 × 24 pixel blocks. Next, the median was taken for each block, the resulting image was then smoothed using a Gaussian filter (sigma = 5) and the image was then resized to match the size of the input image. To apply the correction, experimental FRET ratio images were divided by the ratio correction image.

### Image alignment, background subtraction, segmentation, and speckle filtering

All image analysis methods were conducted using MATLAB. CFP/YFP FRET images were aligned using an alignment algorithm as described [49]. Images were then cropped to ensure equal size. To estimate the background, empty wells containing L-15 media + 2% FBS were imaged with CFP/YFP imaging configurations that were identical to the experimental conditions. Eight empty well frames were collected, and the median was calculated on a per-pixel basis over the image stack. The median well background images were then aligned and cropped to match the size of the experimental images. Next, the background mask was determined for the experimental images. To generate the background mask, the experimental images were log-transformed to enhance the dimmer cell pixels. The image threshold was then calculated using Otsu’s method [50]. The background logical mask was created by finding pixels in the log-transformed image below the threshold. Pixels contained in the background mask were used to find the median intensity in both the empty well image and the experimental image. The ratio of the two median intensities was then used to scale the empty well image to match the intensity of the background pixels in the experimental image. Once scaled, the empty well images were subtracted from the experimental images to remove background.

To segment the cells for further processing, the scaled background image was subtracted from the CFP and YFP images. The CFP and YFP images were added to reduce signal-to-noise ratio, and the sum image was then used to identify the cell mask and the scratch mask. Because the cell pixels were much brighter than the scratch pixels, the dimmest 0.5% of pixels were subtracted from the sum image, and the minimum pixel intensity value was set to 20 prior to log transformation. The log-transformed images were rescaled to a pixel range from the first percentile to the ninetieth percentile, and the background vs foreground threshold was identified using Otsu’s method. Background pixels were identified below the threshold to create a background logical mask. The background mask was morphologically closed to remove small gaps in the mask. Small objects below 50 pixels in area were removed from the mask and small holes in the mask were filled. The inverse of this mask was used to define the cell mask. Both cell and background masks were saved for additional processing.

The raw CFP and YFP images from MCF7 cells had small, but very bright puncta. A speckle filter was developed to remove these puncta from the processed CFP and YFP images. The sum image of the two FRET channels was filtered using a Laplacian operator (alpha = 0.9) to convert the brightest pixels to the smallest negative pixels. The Laplace-filtered image was subtracted from the sum image, effectively making the brightest pixels even brighter. To threshold rare, but bright pixels, the histogram of the image was taken and the intensity value for the first bin with 9 or fewer pixels with positive intensity values was used as the threshold. A bright pixel binary mask was created for all pixels above the bright pixel threshold in the sum image. The bright pixel mask was then morphologically closed to connect neighboring pixels, and objects greater than 300 pixels were removed. Finally, the FRET reporters are plasma membrane-associated, and in some circumstances, were bright enough to be captured by the bright pixel threshold. These objects were more linear as they were essentially tracings of cell edges. To remove these cell membrane objects, the eccentricity for each object was measured. Objects with an eccentricity > 0.6 (more linear than circular) were removed from the mask. The speckle mask was then saved for processing the FRET ratio images.

Finally, to create the FRET ratio images, raw CFP and YFP images were background-subtracted using the scaled background image constructed above. For both CFP and YFP images, pixels in the background mask and pixels in the speckle mask were set to NaNs to remove them from further processing. The background-subtracted and segmented CFP and YFP images were smoothed with a Gaussian filter (sigma = 2) and saved for further processing. Next, the FRET ratio was calculated by dividing the YFP image by the CFP imaged. The ratio correction image described above was then applied to the FRET ratio image. These FRET ratio images were written to movies and exported for later use.

### Computing FRET ratios as a function of distance from the scratch edge

To understand whether cells near the scratch had higher GTPase activity when compared to cells in the monolayer, FRET ratios were measured and binned based on their distance from the scratch edge. To identify the scratch edge, the background masks described above were further analyzed. The scratch is the largest object in the background mask, thus the object with the maximum area was defined as the scratch mask. Next, all other objects in the background mask were removed and the perimeter from the scratch mask was defined. The scratches were consistently generated North to South on the well, thus pixels touching the top and bottom of the perimeter mask image were removed, leaving two scratch edge perimeter masks that corresponded with the left and right sides of the scratch. The scratch edges were then dilated by 2 pixels to ensure overlap with the edge of the cell mask defined above. Additionally, the cell masks were separated based on their relative position to the scratch mask (Left or Right). The MATLAB bwdistgeodesic function was then used to measure the “quasi-euclidean” distance of all pixels in the left and right cell masks based on their distance from the corresponding scratch edge mask. These distance masks were used to sort pixels into 5 µm bins based on their distance from the scratch edge. The ratio correction image was applied to the YFP images, and then the mean intensity values for the background-subtracted and segmented FRET donor and FRET acceptor images were calculated for each bin. The mean FRET ratio for each bin was then calculated. These measurements were compiled for each well within the experimental groups and were used to generate the plots reported.

### Statistical analysis

Prism software (GraphPad Software) was used for all statistical analyses. Statistical significance was determined by two-sided unpaired t test with Welch’s correction, paired t test, Mann–Whitney test, log-rank test, or likelihood ratio test followed by Benjamin–Hochberg correction for multiple hypothesis testing (indicated in the figure legends). *P* values ≤ 0.05 were considered statistically significant.

## Results

### Vangl2-dependent Wnt/PCP signaling promotes breast cancer cell migration regardless of subtype

**Fig. 1:**
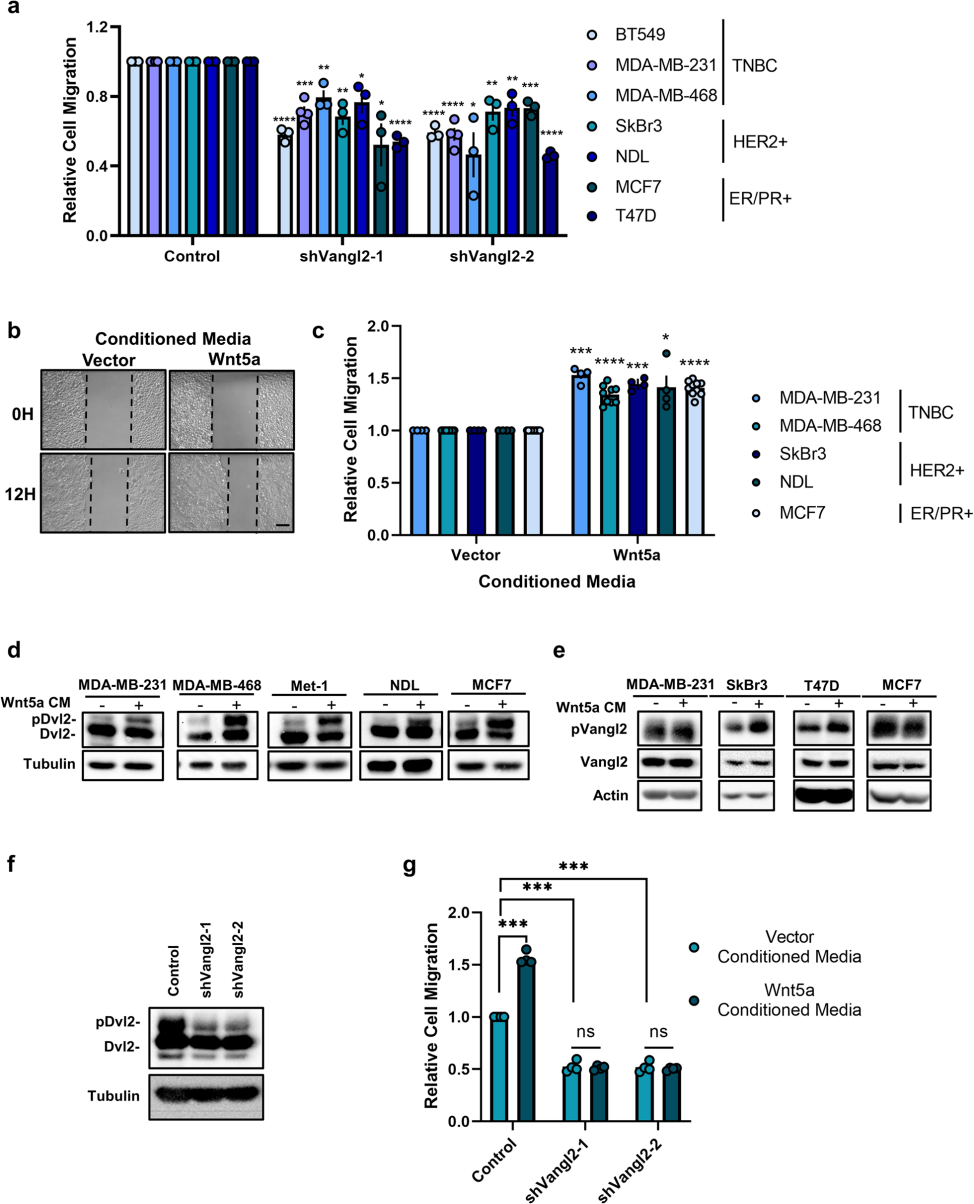
Wnt/PCP signaling mediates breast cancer cell migration. **a** Relative cell migration quantification of BT549, MDA-MB-231,MDA-MB-468, SkBr3, NDL, MCF7, and T47D cells stably expressing Control, shVangl2-1, or shVangl2-2 (BT549 *n* = 3, *p* = 4.81E-05 and *p* = 4.47E-05, MDA-MB-231 *n* = 4, *p* = 0.0001 and *p* = 2.73E-05, MDA-MB-468 *n* = 3, *p* = 0.0078 and *p* = 0.0146, SkBr3 n = 3, *p* = 0.003 and *p* = 0.0057, NDL *n* = 3, *p* = 0.0250 and *p* = 0.0063, MCF7 *n* = 3, *p* = 0.0180 and *p* = 0004, T47D *n* = 3, *p* = 2.13E-05 and *p* = 1.14E-06). **b** Representative images of migrating NDL cells stimulated with Vector- or Wnt5a- conditioned media at 0 and 12 h. Scale bar = 200 µm. **c** Relative cell migration quantification of MDA-MB-231, MDA-MB-468, SkBr3, NDL, and MCF7 cells stimulated with Vector- or Wnt5a-conditioned media (MDA-MB-231 *n* = 4, *p* = 0.0006, MDA-MB-468 *n* = 9, *p* = 2.33E-06, SkBr3 *n* = 4, *p* = 0.0004, NDL *n* = 4, *p* = 0.0344, MCF7 *n* = 10, *p* = 1.54E-08) **d** MCF7, MDA-MB-231, MDA-MB-468, Met-1, and NDL cells stimulated with Vector- or Wnt5a-conditioned media for 1 h blotted for Dvl2**. e** MDA-MB-231, SkBr3, T47D, and MCF7 cells stimulated with Vector- or Wnt5a-conditioned media for 1 h blotted for phospho-Vangl2 and Vangl2. **f** MDA-MB-231 cells stably expressing Control, shVangl2-1, or shVangl2-2 blotted for Dvl2. **g** Relative cell migration quantification of MDA-MB-231 cells stably expressing Control, shVangl2-1, or shVangl2-2 stimulated with Vector- or Wnt5a- conditioned media (control: vector- vs Wnt5a-conditioned media *n* = 4, *p* = 0.0003, Control vs shVangl2-1 + vector-conditioned media *n* = 4, *p* = 0.0003, control vs shVangl2-2 + vector-conditioned media *n* = 4, *p* = 0.0003, shVangl2-1: vector- vs Wnt5a-conditioned media *n* = 4, *p* = 0.7256, shVangl2-2: vector- vs Wnt5a-conditioned media *n* = 4, *p* = 0.5804). Bar graphs represent the mean ± sem of experimental replicates (*n*). Significance was determined by a two-sided unpaired *t* test with Welch’s correction, *p < 0.05, **p < 0.01, ***p < 0.001, ****p < 0.0001

Expression analysis of patient-invasive breast tumors suggests that Vangl2 dysregulation is a feature common to breast tumors rather than being associated with a single molecular subtype [31]. Consistently, we find that Vangl2 expression is highly heterogeneous across a panel of human and murine breast cancer cell lines encompassing several molecular subtypes as well as tumors from two commonly employed genetic models of murine mammary carcinoma (Additional file 4: Fig. S1a–f). However, previous studies have focused on triple-negative breast cancers (TNBC) or basal subtypes [28, 31], leaving the contribution of Vangl to migration and invasion of the majority of breast cancer subtypes unexplored. Therefore, we interrogated whether loss of Vangl2 impacts cell motility of diverse breast cancer cells of distinct molecular subtypes: triple-negative BT549, MDA-MB-231 and MDA-MB-468, HER2-positive SkBr3 and NDL, and ER/PR-positive MCF7 and T47D. Cells were transduced with lentivirus encoding *VANGL2*-targeted shRNAs and knockdown was confirmed by *q*PCR (Additional file 4: Fig. S1 g). We observed that loss of *VANGL2* expression significantly impairs breast cancer cell migration, indicated by the reduced ability of *VANGL2* knockdown cells to migrate into a scratch made in the cellular monolayer (Fig. 1a). These data demonstrate that Vangl2 is critical to efficient breast cancer cell motility regardless of breast cancer subtype.

In motile cells, activation of Vangl-dependent Wnt/PCP signaling occurs by binding of a non-canonical Wnt ligand such as Wnt5a to Fzd receptors at the plasma membrane, resulting in recruitment and phosphorylation of Dvl [51]. Transmembrane proteins Vangl1 and Vangl2 and activated Dvl may serve as both scaffolds and activators of downstream effector components that mediate context- and tissue-specific actin cytoskeletal rearrangements to promote cellular motility [32]. Consistent with previous reports [26], we found that Wnt5a is a potent activator of Wnt/PCP signaling that drives breast cancer cell migration. Stimulation of breast cancer cell lines with Wnt5a-conditioned media enhances cellular migration (Fig. 1b, c) and robustly increases phosphorylation of Dvl2 (Fig. 1d and Additional file 4: Fig. S2a) compared to vector control-conditioned media. Similar to Vangl2 (Additional file 4: Fig. S1a, b), Wnt5a ligand expression is heterogeneous among breast cancer cell lines and genetic models of murine mammary carcinoma (Additional file 4: Fig. S2b–c). To exclude the possibility that Wnt5a activates the canonical Wnt pathway in our experiments, we assessed phosphorylation of β-catenin, a marker of canonical Wnt signaling [52]. Stimulation of breast cancer cells with Wnt5a-conditioned media does not significantly impact β-catenin phosphorylation, whereas stimulation with media conditioned with the potent canonical Wnt activating ligand Wnt3a significantly reduces β-catenin phosphorylation (Additional file 4: Fig. S2d–e). Thus, Wnt5a-dependent migration and Dvl2 phosphorylation in breast cancer cells is driven by engagement of a non-canonical Wnt signaling pathway rather than canonical Wnt signaling.

Dvl2 phosphorylation is also a common feature of other non-canonical Wnt pathways independent of Wnt/PCP signaling [53], and while a downstream effector specific to Wnt/PCP signaling has yet to be identified, this branch of non-canonical Wnt signaling requires the formation of Vangl-dependent complexes [18]. Importantly, Vangl2 phosphorylation at specific residues is required for Vangl2 function in mammalian Wnt/PCP signaling [54]. Consistent with findings from mammalian development [54], Wnt5a stimulation phosphorylates Vangl2 at critical functional residues across a panel of breast cancer cell lines (Fig. 1e and Additional file 4: Fig. S2f) and loss of *VANGL2* suppresses Dvl2 phosphorylation (Fig. 1f). We determined that Wnt5a-mediated migration is ablated in *VANGL2* knockdown breast cancer cells (Fig. 1g), demonstrating that Wnt5a specifically activates Vangl-dependent Wnt/PCP signaling in breast cancer cells.

### High Vangl expression aberrantly engages Wnt/PCP signaling and enhances breast cancer cell motility

**Fig. 2:**
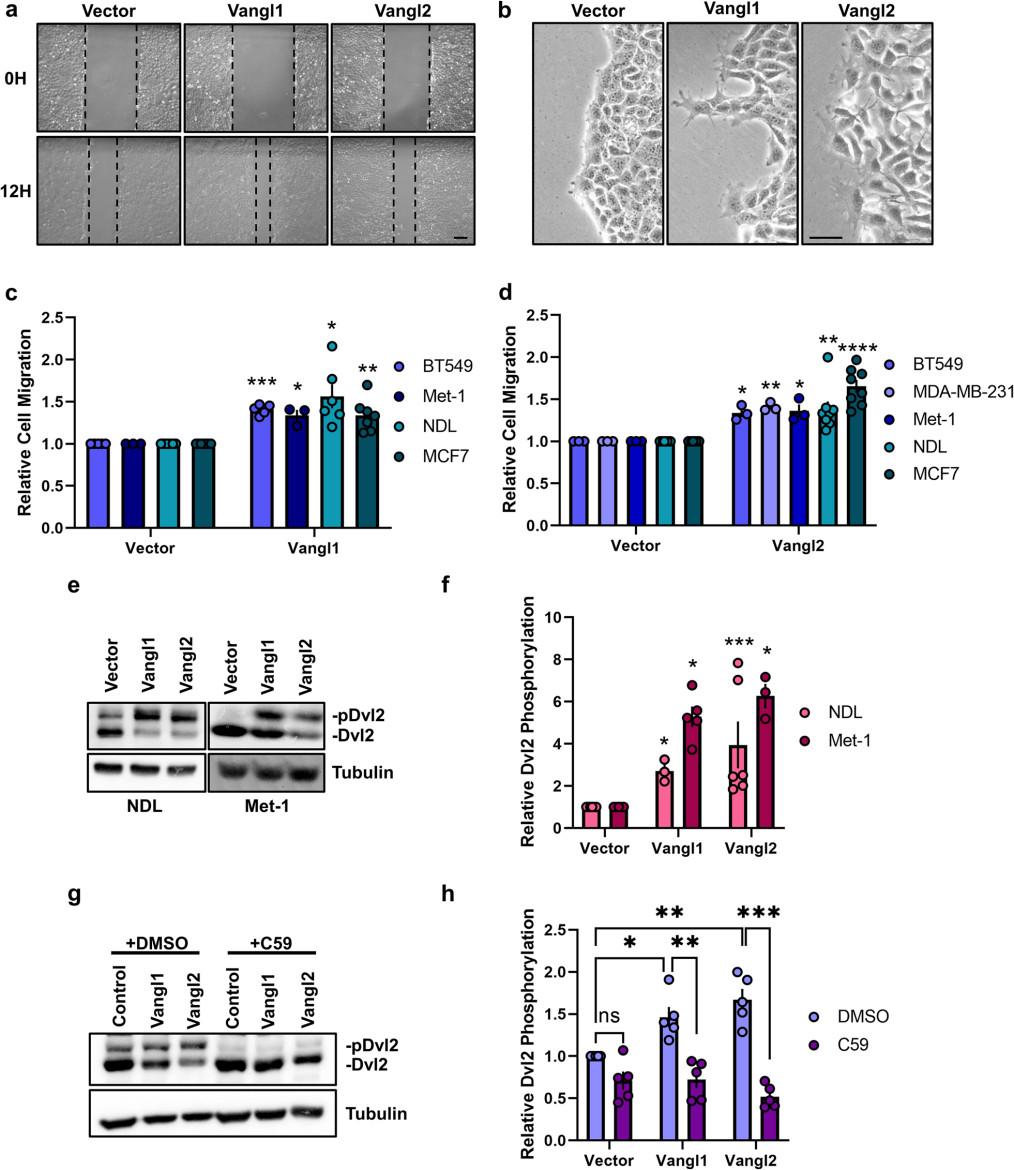
Vangl1 and Vangl2 overexpression promote breast cancer cell migration and potentiate Wnt/PCP signaling. **a**, **b** Representative brightfield images of migrating NDL cells stably expressing Vector, Vangl1, or Vangl2 at 0 and 12 h, scale bar = 200 µm (**a**) and leading-edge dynamics at 12 h, scale bar = 50 µm (**b**). **c** Relative cell migration quantification of BT549, Met-1, NDL and MCF7 cells stably expressing Vector or Vangl1 (BT549 *n* = 5, *p* = 0.0001, Met-1 *n* = 3, *p* = 0.0339, NDL *n* = 6, *p* = 0.0108, MCF7 *n* = 7, *p* = 0.0035,). **d** Relative cell migration quantification of BT549, MDA-MB-231, Met-1, NDL, and MCF7 cells stably expressing Vector or Vangl2 (BT549 *n* = 3, *p* = 0.0210, MDA-MB-231 *n* = 3, *p* = 0.0050, Met-1 *n* = 3, *p* = 0.0412, NDL *n* = 8, *p* = 0.0049, MCF7 *n* = 8, *p* = 6.17E-05). **e–f** NDL and Met-1 cells stably expressing Vector, Vangl1 or Vangl2 blotted for Dvl2 (**e**) and quantification of Dvl2 phosphorylation in NDL and Met-1 cells stably expressing Vector, Vangl1 or Vangl2 (**f**) (NDL-Vangl1 *n* = 3, *p* = 0.0291, NDL-Vangl2 *n* = 6, *p* = 0.0453, Met-1-Vangl1 *n* = 5, *p* = 0.0009, Met-1-Vangl1 *n* = 3, *p* = 0.0119). **g**,** h** NDL cells stably expressing Vector, Vangl1, or Vangl2 treated with DMSO or 100 nM C59 for 24 h blotted for Dvl2 (**g**) and quantification of relative Dvl2 phosphorylation (**h**). Bar graphs represent the mean ± sem of experimental replicates (*n*). Significance was determined by a two-sided unpaired *t* test with Welch’s correction, *p < 0.05, **p < 0.01, ***p < 0.001, ****p < 0.0001

Our observations that Vangl2 mediates breast cancer migration and is required for Wnt5a-mediated cell migration (Fig. 1), combined with previous reports that elevated *VANGL2* expression correlates with worsened metastasis-free survival in breast cancer patients [31], suggest that high Vangl expression may result in enhanced cellular migration and aberrant engagement of Wnt/PCP signaling in breast tumors. To investigate this possibility, we stably overexpressed Vangl1 or Vangl2 via lentiviral infection in human and mouse breast tumor cell lines (Additional file 4: Fig. S3a–d). Breast cancer cells overexpressing Vangl1 or Vangl2 exhibit increased motility and Dvl2 phosphorylation compared to cells transduced with control lentivirus (Fig. 2a, c–f) and display a distinctive hyper-protrusive leading-edge morphology (Fig. 2b). These observations indicate that Vangl proteins are sufficient to aberrantly engage Wnt/PCP signaling either independent of Wnt ligand or by potentiating signaling from endogenous Wnt ligand.

To distinguish between these possibilities, we treated Vangl overexpressing breast cancer cells with the Porcupine antagonist C59, which impairs palmitoylation and subsequent secretion of Wnt ligands [55], to deplete endogenous Wnt5a ligand. C59 treatment resulted in ablation of Vangl-mediated Dvl2 phosphorylation (Fig. 2g, h), demonstrating that aberrant Wnt/PCP signaling mediated by Vangl overexpression is Wnt ligand-dependent. Taken together, these findings suggest that activation of Wnt/PCP signaling in breast cancer cells, accomplished either by exposing cells to elevated levels of non-canonical Wnt ligand or by potentiating signals from endogenous Wnt ligands through overexpression of Vangls, enhances cellular motility.

### Wnt/PCP signaling drives breast carcinoma cell collective invasion

Collective cell invasion is driven by leader cells that aggressively invade while remaining attached to follower cells, resulting in the formation of contiguous invasive strands [6, 11, 56]. Invasive leader cells are molecularly and behaviorally distinct from bulk tumor cells, and in some mammary tumor models and human breast tumors express the basal epithelial marker cytokeratin 14 (K14) [56]. Importantly, K14-positive leader cells are not enriched for markers of stemness or EMT [56], underscoring the unique character of this population and distinguishing these cells from classically defined invasive mediators of metastasis.

To investigate the contribution of Wnt/PCP signaling to breast cancer cell collective invasion, we employed an ex vivo 3D collagen invasion assay [46], in which tumor organoids derived from the highly aggressive, metastatic *MMTV-PyMT* mouse model [57, 58] form multicellular epithelial cell protrusions that invade a collagen matrix. Here, individual *MMTV-PyMT* tumor cells were first seeded in Matrigel, transduced with lentivirus encoding Wnt/PCP components (Additional file 4: Fig. S4a,c), and cultured for one week to generate tumor organoids. Organoids were then transferred to 3D collagen I gels, a model for the microenvironment surrounding invasive breast cancers [46], and a fraction of epithelial cells became K14-positive leader cells that formed multicellular protrusions of collectively invading cells upon stimulation with *b*FGF [56].

**Fig. 3:**
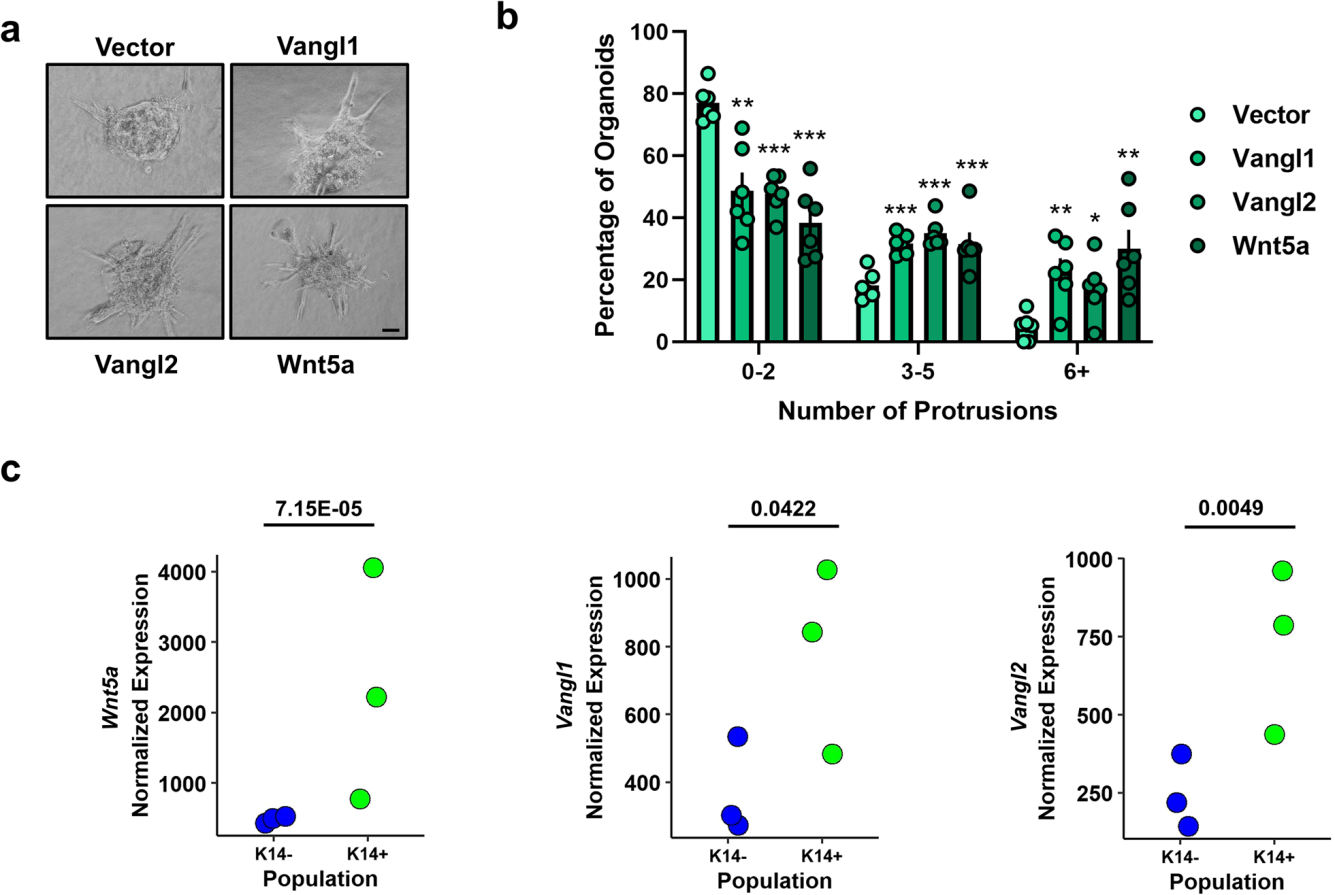
Wnt/PCP signaling drives collective cell invasion ex vivo and is upregulated in the K14-positive leader cell population. **a** Representative bright field images of *MMTV-PyMT*-derived mouse mammary tumor organoids stably overexpressing Vector, Vangl1, Vangl2 or Wnt5a invading into collagen in the presence of 2.5 nm *b*FGF, scale bars = 50 µm. **b** Quantification of the percentage of organoids counted with 0–2, 3–5, and 6 + collectively invading protrusions for Vector-, Vangl1-, Vangl2-, or Wnt5a-expressing organoids (Vector *n* = 547, Vangl1 *n* = 371, Vangl2 *n* = 276, Wnt5a *n* = 456 organoids counted from six independent experiments, *p* values represent Vector (V) vs Vangl1 (V1), Vangl2 (V2), Wnt5a (W), 0–2 protrusions; V vs V1 *p* = 0.0011, V vs V2 *p* = 6.32E-06, V vs W *p* = 2.50E-05, 3–5 protrusions; V vs V1 *p* = 0.0001, V vs V2 *p* = 8.94E-05, V vs W *p* = 0.0092, 6 + protrusions; V vs V1 *p* = 0.0027, V vs V2 *p* = 0.0132, V vs W *p* = 0.0024). Bar graphs represent the mean ± sem of experimental replicates (*n*), significance was determined by a two-sided unpaired *t* test, *p < 0.05, **p < 0.01, ***p < 0.001, ****p < 0.0001. **c** Analysis of RNA-sequencing data set SRP066316 from NCBI Sequence Read Archive for *Wnt5a, Vangl2,* and *Vangl1* transcript in K14- and K14 + cells derived from *MMTV-PyMT* tumors (*Wnt5a p* = 7.15E-05, *Vangl2 p* = 0.0049, *VANGL1 p* = 0.0422), significance was determined by likelihood ratio test followed by Benjamin–Hochberg correction for multiple hypothesis testing

We observed that lentiviral-mediated overexpression of Wnt5a, Vangl1, or Vangl2 significantly increases the frequency of *b*FGF-dependent collectively invading strands formed by *MMTV-PyMT* organoids (Fig. 3a, b, Additional file 4: Fig. S4d). Expression of Wnt/PCP components was not sufficient to stimulate collective invasion in the absence of *b*FGF (Additional file 4: Fig. S4e), indicating that Wnt/PCP signaling cooperates with additional signaling pathways to promote collective invasion rather than independently driving the formation of invasive protrusions. In support of our findings, analysis of a publicly available dataset that accompanied the foundational study describing the contributions of K14-positive leader cells to breast cancer progression [6] determined that *Wnt5a*, *Vangl1,* and *Vangl2* transcripts are significantly elevated in the K14-positive tumor cell population (Fig. 3c). Other Wnt/PCP component transcripts including non-canonical Frizzled receptors and Dvl were not significantly altered (Additional file 4: Fig. S5). These data demonstrate that key Wnt/PCP components are highly expressed in K14-positive leader cells at the tip of invading strands compared to K14-negative follower cells or non-invading tumor organoid cells, indicating that Wnt/PCP signaling specifically augments the protrusive activity of K14-positive leader cells that drive collective invasion.

### Vangl localizes to the leading edge of collectively migrating breast cancer cells and promotes a hyper-protrusive leading edge

Wnt/PCP signaling is essential for the establishment and maintenance of polarity in epithelial tissues, where it regulates cell polarization in the planar axis across the surface of an epithelial sheet [59]. Planar polarity across the tissue is achieved through the asymmetric distribution of core Wnt/PCP complexes within individual cells reinforced by intracellular antagonism between opposing complexes [60–63]. Intercellular complexes formed by opposing complexes on adjacent cells then transmit that asymmetry to neighboring cells [64–66], and propagation of this asymmetry across many cell distances allows for the integration of global cues to locally polarized cellular behavior [59]. Wnt/PCP signaling is also vital to collective cell motility events critical to embryonic tissue patterning [12, 18]. The requirement for Wnt/PCP complex asymmetry in collectively migrating cells has, however, remained unclear, despite significant efforts to understand component localization in migrating breast cancer cells [27–29].

**Fig. 4:**
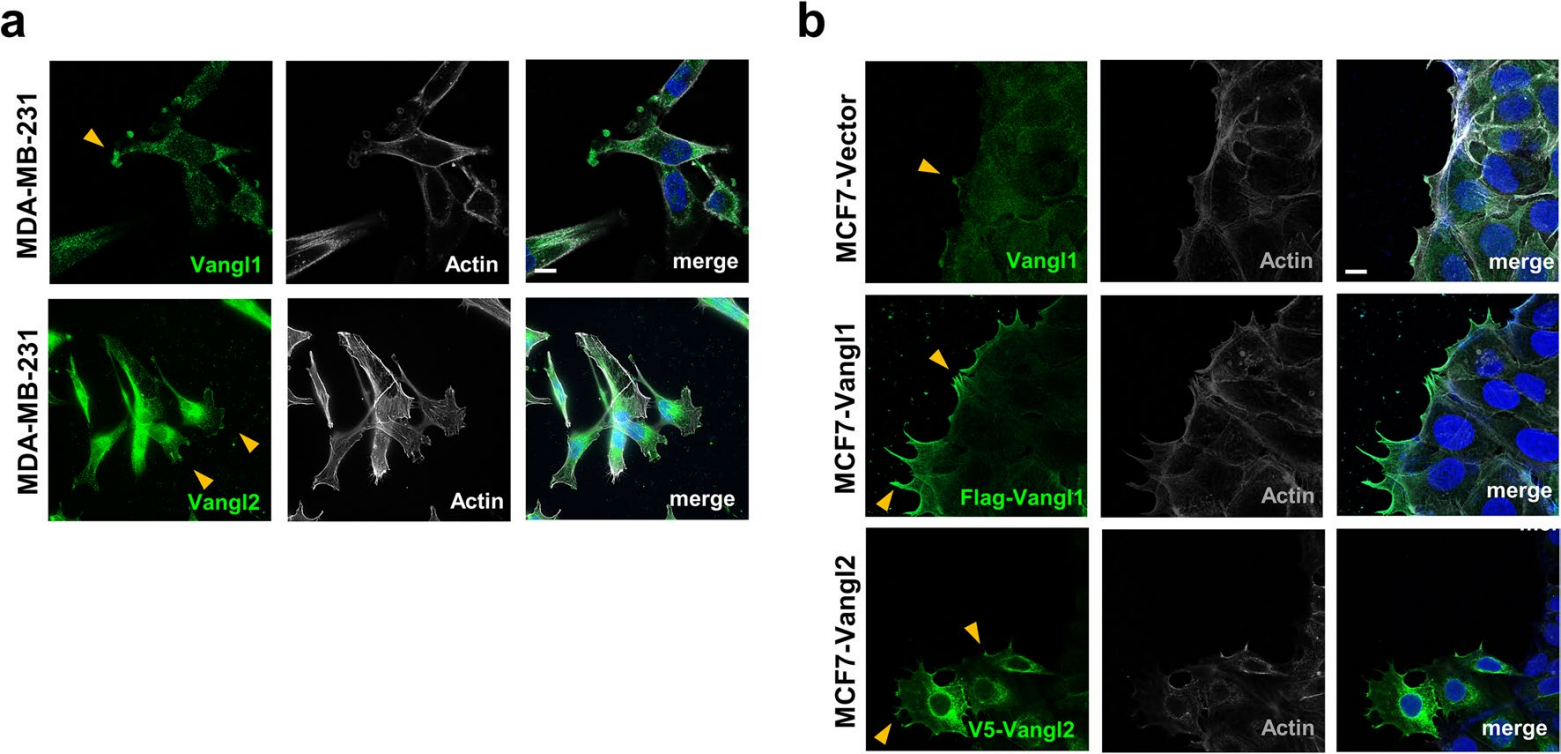
Vangl localizes to the migratory protrusions of single migratory cells and the leading edge of leader cells in collectively migrating breast cancer cells and promotes a hyper-protrusive morphology. **a** Representative confocal images of migratory MDA-MB-231 cells stained for Vangl1:green or Vangl2:green, Actin: gray, and DAPI: blue. **b** Representative confocal images of collectively migrating MCF7-Vector cells stained for Vangl1: green, Actin: gray, and DAPI: blue, MCF7-Flag-Vangl1 cells stained for Flag: green, Actin: gray, and DAPI: blue, and MCF7-V5-Vangl2 cells stained for V5: green, Actin: gray, and DAPI: blue, yellow arrows: Vangl-rich protrusions, scale bar = 10 µm

We employed immunofluorescence microscopy to assess the localization of endogenous and ectopically expressed Wnt/PCP components Vangl1 and Vangl2 in singly and collectively migrating breast cancer cells. We observe Vangl1 and Vangl2 localization to the leading edge of migrating MDA-MB-231 breast cancer cells within actin-rich migratory protrusions (Fig. 4a). MCF7 breast cancer cells were employed as a model of collective cell migration based on our observations that they both migrate as cohesive sheets with E-cadherin-rich cell–cell junctions (Additional file 4: Fig. S6). In MCF7 cells, we observed that both Vangl1 and Vangl2 localize to actin-rich migratory protrusions in cells at the leading edge of a collectively migrating cohort (Fig. 4a). Consistent with observations that Vangl1 or Vangl2 overexpression enhances cellular motility (Fig. 2c–d), Vangl1 or Vangl2 overexpression in MCF7 breast cancer cells elicits a hyper-protrusive leading edge (Fig. 4b), suggesting that elevated Vangl may mediate the assembly of Wnt/PCP complexes that promote the formation of pro-migratory protrusions that drive collective cell migration. Indeed, elevated Vangl1 expression significantly increases both the number of Vangl1-rich protrusions in leader cells and the percentage of leader cells with Vangl1-rich protrusions in a collectively migrating sheet of MCF7 breast cancer cells (Additional file 4: Fig. S6b–c). These data suggest that Vangl mediates the formation of pro-migratory protrusions in single migratory cells as well as leader cells of collectively migrating breast cancer cohorts and that high Vangl expression drives enhanced cellular invasiveness through the regulation of aberrant leading-edge protrusion formation.

### Vangl2 regulates RhoA activity in leader cells of collectively migrating breast cancer cells

Our findings that Vangl drives collective cell motility and invasion as well as mediates the formation of pro-migratory protrusions in leader cells of collectively migrating breast cancer cells led us to question the molecular mechanisms by which Vangl achieves these outcomes. We hypothesized that Vangl may regulate the actin cytoskeleton in leader cells via engagement of Rho GTPases Rac1 and RhoA, which are engaged in Wnt/PCP-mediated motility during vertebrate gastrulation in developing embryos [67, 68]. The regulation of Rac1 and RhoA GTPase activity is complex and permits context-specific activation of signaling events at specific subcellular localizations with precise kinetics [69]. Unfortunately, previous studies that investigated the ability of Wnt/PCP components to specifically engage and regulate Rho GTPases in cancer cells have predominantly assessed global GTPase activity in lysed cells via GST pull-down assays [23, 24, 32], leaving both the localization and kinetics of Wnt/PCP-mediated RhoA and Rac1 activity and the contribution of Wnt/PCP components to leader cell biology largely unexplored.

**Fig. 5:**
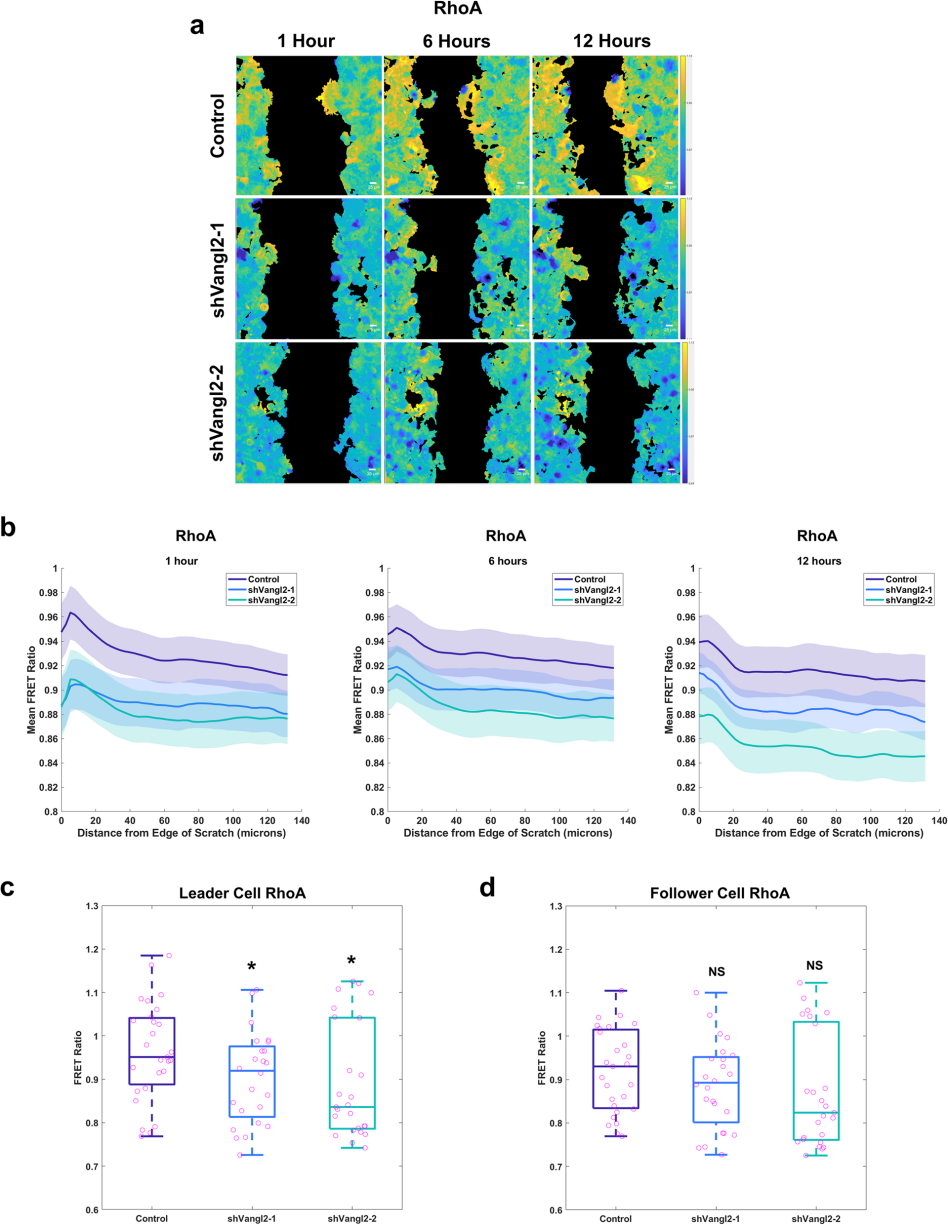
Vangl2 regulates RhoA activity in leader cells of collectively migrating breast cancer cells. **a** Representative spatial activity profiles of RhoA in collectively migrating MCF7 cells stably expressing RhoA-FRET biosensor and Control (top row), shVangl2-1 (middle row), or shVangl2-2 (bottom row) at 1 h (left column), 6 h (center column), and 12 h (right column. Color bars indicate the range of RhoA-FRET biosensor ratios. Scale bar = 25 µm. **b** RhoA activity as a function of distance in µm from the leading edge of collectively migrating MCF7 cells stably expressing Vector (*n* = 27 wells), shVangl2-1 (*n* = 24 wells), or shVangl2-2 (*n* = 25 wells) at 1, 6, and 12 h of migration, error bars indicate ± sem. **c**, **d** RhoA activity after 1 h of migration at 5 µm (**c**) and 100 µm (**d**) from the leading edge of collectively migrating MCF7 cells stably expressing Vector, shVangl2-1 (Vector vs shVangl2-1, 5 µm* p* = 0.00388, 100 µm *p* = 0.1010, or shVangl2-2 (Vector vs shVangl2-2, 5 µm *p* = 0.0384, 100 µm *p* = 0.1309), significance was determined by a two-sided unpaired *t* test

We investigated Wnt/PCP-mediated spatiotemporal dynamics of GTPase signaling in real time via time-lapse imaging in collectively migrating breast cancer cells by monitoring GTPase activity using stably expressed Rac1 or RhoA fluorescence resonance energy transfer (FRET) biosensors [49]. Here, MCF7 cells stably expressing non-targeting control shRNA or two independent *VANGL2*-targeted shRNAs and a Rac1 or RhoA biosensor were seeded onto glass-bottom plates, the confluent monolayer was scratched at zero hours, and scratches imaged every fifteen minutes throughout the 12-h migration assay (Fig. 5a, Additional files 1, 2, 3: Videos S1–S3). The mean FRET ratio, which indicates Rac1 or RhoA activity, was measured after 1, 6, and 12 h of migration and plotted as a function of distance from the leading edge of the migrating cohort of MCF7 cells using a custom MATLAB script to quantify Rac1 and RhoA activity across the monolayer of cells (Fig. 5b, Additional file 4: Fig. 7a). Briefly, our MATLAB script identified the leading edge of the migrating cohort of MCF7 cells (Additional file 4: Fig. S7b) and binned migrating cells based on their distance from the edge of the scratch (Additional file 4: Fig. S7c).

Spatial analysis revealed that RhoA activity is highest approximately 5–10 µm from the edge of the scratch (Fig. 5b) in a collectively migrating cohort after 1 h. Indeed, RhoA activity is significantly higher 5 µm from the edge of the scratch as compared to cells 100 µm from the edge of the scratch after 1 h of migration (Additional file 4: Fig. S7d). MCF7 cells are roughly 20–25 µm in diameter; thus, the elevated RhoA near the scratch edge likely represents leader cells. Depletion of *VANGL2* significantly reduced RhoA activity in leader cells 5 µm from the edge of the scratch (Fig. 5c) and appeared to reduce RhoA activity in follower cells 100 µm from the edge of the scratch, but did not pass our threshold for statistical significance (Fig. 5d) after 1 h of migration. Rac1 signaling did not differ spatially within the migrating cohort and modulation of *VANGL2* did not reproducibly alter Rac1 activity (Additional file 4: Fig. S7a). Collectively, these findings suggest that Vangl2-dependent Wnt/PCP signaling specifically regulates RhoA activity in leader cells to support actin cytoskeletal rearrangements critical to the formation of pro-migratory protrusions that drive collective migration.

### Vangl2 deletion suppresses mammary tumor metastasis to the lungs but does not alter primary tumor growth

Collectively, our in vitro and ex vivo findings demonstrate that Vangl is a critical mediator of breast cancer cell migration and collective invasion, processes critical to successful metastatic dissemination. However, the contribution of Vangl to breast tumor metastasis has never been reported. We assessed the functional importance of Vangl2 to mammary tumorigenesis and tumor cell metastatic dissemination by specifically ablating *Vangl2* in the mammary epithelium of *MMTV-NDL* mice. In this well-characterized genetically engineered mouse model of breast cancer, an activated ErbB2 mutant encoded by the transgenic rat *c-ErbB2/neu* allele under the control of the MMTV promoter drives the formation of metastatic multifocal mammary tumors at approximately 20 weeks of age [38] (Additional file 4: Fig. S8a). The *MMTV-NDL* murine model was selected based on our observations that Vangl2 is highly expressed in tumors from this model (Additional file 4: Fig. S1b) and Vangl2 overexpression was observed in breast cancer patient tumors of all molecular subtypes [31]. Effective deletion of *Vangl2* in mammary tumors of *Vangl2*^*fl/fl*^*;MMTV-Cre*^±^*;MMTV-NDL*^±^ (Vangl2^fl/fl^/NDL) mice relative to *Vangl2*^*fl/fl*^*;MMTV-NDL*^±^ (Vangl2^+/+^/NDL) mice was confirmed by *q*PCR (Additional file 4: Fig. S8b). Although *Vangl1* may compensate for loss of *Vangl2* in some contexts [18], *Vangl1* transcript is not significantly altered in Vangl2^fl/fl^/NDL mammary tumors relative to Vangl2^+/+^/NDL tumors (Additional file 4: Fig. S8c). *Vangl2* ablation did not produce discernable effects on viability, breeding, or lactation, and no differences in mammary gland architecture were noted between genotypes in adult virgin mammary glands (Additional file 4: Fig. S8d).

**Fig. 6:**
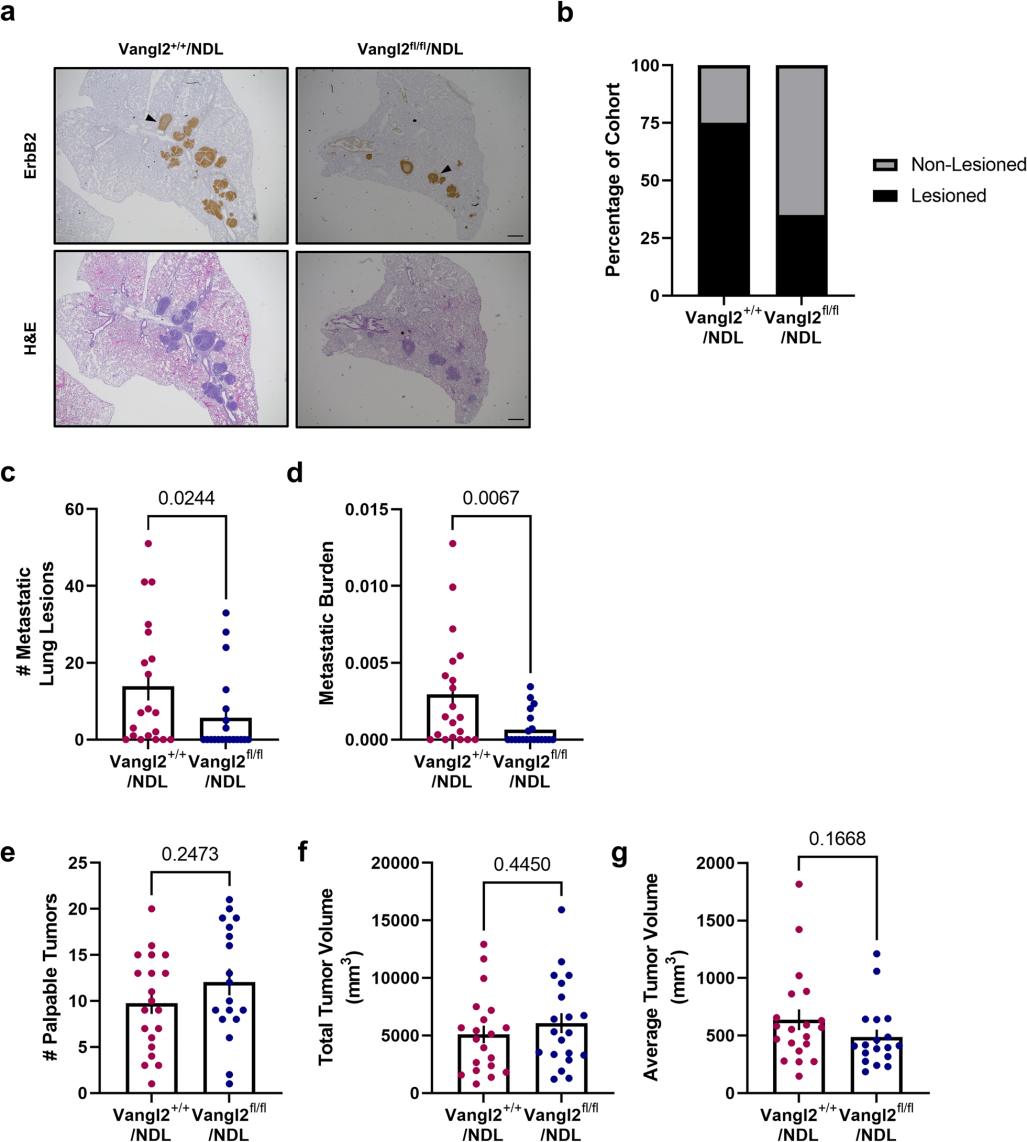
Vangl2 deletion suppresses mammary tumor metastasis to the lung. **a** Representative images of formalin-fixed, paraffin-embedded sections from Vangl2^+/+^/NDL and Vangl2^fl/fl^/NDL lung tissue following immunodetection of ErbB2 (top panel) and H&E staining (bottom panel). Examples of ErbB2-positive metastatic lung lesions are denoted by black arrowheads, scale bar = 500 µm. **b**–**d** Lung lobes (5 lobes per mouse) were evaluated by histology for the occurrence of metastatic lesions for Vangl2^+/+^/NDL (*n* = 20) and Vangl2^fl/fl^/NDL (*n* = 20) tumor-bearing animals. The number of mice bearing metastatic lesions (**b**), numbers of metastatic lesions (**c**), and metastatic burden (**d**) were assessed. **e–g** Vangl2/NDL primary tumor growth characteristics were assessed for Vangl2^+/+^/NDL (*n* = 20) and Vangl2^fl/fl^/NDL (*n* = 20) tumor-bearing animals, including number of palpable tumors (**e**), total tumor volume (**f**), and average tumor volume (**g**). Significance was determined by Mann–Whitney test and bar graphs represent the mean ± sem of experimental replicates (*n*)

Despite similar kinetics of primary tumor initiation and growth in Vangl2^+/+^/NDL and Vangl2^fl/fl^/NDL mice (Additional file 4: Fig. S9a–d), *Vangl2*-depleted tumors are significantly less metastatic than *Vangl2*-intact tumors (Fig. 6a–d). Analysis of lung tissue revealed that deletion of *Vangl2* in *MMTV-NDL* tumors results in significantly reduced frequency of metastatic disease (Fig. 6b), number of lung metastases (Fig. 6c), and overall metastatic burden (Fig. 6d) despite similar primary tumor characteristics such as numbers of palpable tumors (Fig. 6e), total tumor volume (Fig. 6f), average tumor volume (Fig. 6g), tissue histology (Additional file 4: Fig. S9e–f), proliferative capacity (Additional file 4: Fig. S9 g-h), and apoptosis (Additional file 4: Fig. S9i–j). Importantly, *Vangl2* appears to be critical to successful metastatic dissemination to the lungs (Fig. 6c–d) but is not required for the proliferation of metastatic lesions in Vangl2^+/+^/NDL and Vangl2^fl/fl^/NDL mice (Additional file 4: Fig. S10a–b). Further, cells derived from Vangl2^+/+^/NDL and Vangl2^fl/fl^/NDL tumors injected into the tail veins of FvB/NJ mice exhibited no differences in metastatic lesion colonization efficiency (Additional file 4: Fig. S10c–f). Taken together, these findings may suggest that the reduced incidence of metastasis observed upon *Vangl2* ablation is the result of reduced dissemination from the primary tumor rather than suppressed outgrowth of colonies in the lungs.

Collectively, our observations demonstrate for the first time that Vangl2-mediated Wnt/PCP signaling is critical to metastatic dissemination of breast tumor cells, likely through the promotion of local collective cell migration and invasion from the primary tumor. Mechanistically, our observations demonstrate that Vangl acts at the leading edge of collectively migrating cells to prompt the RhoA-dependent cytoskeletal dynamics necessary for pro-migratory cellular protrusion formation. By extension, we propose that the high Vangl expression frequently observed in breast cancer patient primary tumors drives aberrant Vangl-dependent Wnt/PCP signaling, resulting in a hyper-protrusive leading edge that supports invasiveness and successful metastatic dissemination.

## Discussion

Metastatic disease is responsible for the majority of cancer-related deaths [70], despite significant investment into elucidating molecular drivers of metastasis and identifying opportunities for therapeutic intervention. The acquisition of migratory and invasive behaviors by tumor epithelial cells is a critical step for metastatic dissemination, but the molecular mechanisms underlying this transition remain incompletely described. In this study, we demonstrate that the Wnt/PCP-specific transmembrane scaffold protein Vangl2 is vital for the efficient formation of metastatic lung lesions from ErbB2-driven mammary tumors, mediates collective cell invasion in conjunction with the permissive factor bFGF in PyMT-driven mammary tumor organoids, and is highly expressed in invasive leader cells where it appears to mediate pro-migratory protrusive activity via regulation of RhoA.

Despite reports that Vangl2 is highly expressed in 25% of invasive breast cancers [18] and that elevated *VANGL2* correlates with advanced-stage disease and decreased metastasis-free survival of breast cancer patients [31], the functional role of Vangl2 in breast cancer malignancy has remained largely unexplored and its contribution to breast cancer metastasis has not been reported. Here, we present the novel finding that Vangl2, a central component of Wnt/PCP signaling, is critical for the metastasis of *MMTV-NDL* tumors but dispensable for both the initiation and growth of primary tumors. Our observations are consistent with a role for Vangl2 in mediating cell motility events critical to developing embryos [12, 59], but conflict with a previous report in which shRNA-mediated knockdown of *VANGL2* decreased proliferation of SUM159 and HCC1806 breast cancer cells xenografted into the flanks of NSG mice [31]. Based on reports that *VANGL2* upregulation is associated with higher-grade breast tumors [31], we hypothesize that elevated *VANGL2* expression and resulting activation of Wnt/PCP signaling is a feature of late-stage or advanced disease. Because the autochthonous *MMTV-NDL* model recapitulates the full course of breast cancer development beginning from an untransformed mammary epithelial cell, Wnt/PCP signaling may have not yet been aberrantly engaged for the majority of *MMTV-NDL* primary tumor growth. However, xenograft tumors derived from cell line models of late-stage breast cancer, may more heavily rely upon already aberrantly engaged Wnt/PCP signaling for primary tumor growth. This discrepancy, coupled with observations that Vangl2 is aberrantly expressed across diverse subtypes of invasive breast tumors [31], suggests that Wnt/PCP signaling may be a marker that could be used clinically to predict invasive or aggressive breast cancer.

While the loss of *Vangl2* significantly reduces both metastatic burden and number of metastatic lesions, it does not appear to impact the efficiency with which disseminated tumor cells colonize the lung nor the proliferation of established metastatic lesions, suggesting that Vangl2 may be mediating local migration and invasion from the primary tumor. This is supported by our findings that loss of Vangl2 suppresses migration in vitro while high Vangl expression drives cell migration in vitro and collective cell invasion ex vivo*.* Elevated *Vangl1* and *Vangl2* expression in K14-positive invasive leader cells in vivo suggests that Vangl may be important to leader cell biology. Our novel finding that high Vangl1 or Vangl2 expression is sufficient to mediate phosphorylation of Dvl2, a critical downstream effector of Wnt/PCP signaling, led us to speculate that Vangl-mediated Wnt/PCP signaling is critical in promoting the cytoskeletal remodeling necessary for the invasive behavior of leader cells during collective migration and invasion.

Our findings that high expression of Vangl1 or Vangl2 results in a hyper-protrusive leading edge suggest that Vangl localizes to the protrusive leading-edge membrane of leader cells in migrating breast cancer cell cohorts to regulate the cytoskeleton. The localization of Wnt/PCP component Vangl in collectively migrating cells is unresolved [27, 28], and questions remain regarding whether classic Wnt/PCP component asymmetry observed in polarized epithelial tissues is conserved in migrating cells [11]. We observed that Vangls localize to the leading edge of singly and collectively migrating breast cancer cells and that high expression of Vangl1 or Vangl2 in leader cells results in a hyper-protrusive leading edge. To assess whether Vangl regulates the cytoskeleton in leader cells, we employed time-lapse microscopy of collectively migrating breast cancer cells depleted of Vangl2 and stably expressing FRET biosensors for cytoskeletal regulators Rac1 and RhoA [68]. We found that RhoA activity is consistently elevated in leader cells, whereas Rac1 has a more uniform spatial activity pattern across both leader and follower cell populations, which is consistent with previous studies of collectively migrating epithelial cells [71, 72]. Loss of Vangl2 significantly reduces RhoA activity in leader cells, but has no impact on follower cell RhoA activity, suggesting that a Vangl2-RhoA signaling axis may be specific to leader cell biology and responsible for the protrusive leading-edge phenotype observed in Vangl overexpressing breast cancer cells.

Overall, our findings provide substantive insight into the mechanism by which Vangl proteins specifically mediate the formation of pro-migratory protrusions at the leading edge of leader cells in collectively migrating cohorts. Additionally, our observations suggest that Vangl-mediated regulation of RhoA dynamics in leader cells is critical to Wnt/PCP-mediated collective cell migration and invasion. However, the molecular underpinnings of Vangl-mediated RhoA activity within leader cells are not clear. In vertebrate gastrulation, Wnt/PCP signaling appears to drive cellular motility via engagement of Rho family GTPases in a manner that depends on both the cytoplasmic effector Dvl and Daam1, a Formin homology protein that mediates Wnt-induced Dvl-Rho complexes [67]. While this study did not investigate whether Vangl is a required component of this complex, we speculate that Vangl may serve as a required scaffold upon which the Dvl-Daam1-RhoA complex assembles in leader cells. This is consistent with previous reports suggesting that Vangl may be a master scaffold upon which diverse complexes assemble [28, 31, 32]. These findings, coupled with our observations that Vangl localizes to leader cells in collectively migrating and invading cohorts and drives the formation of pro-migratory protrusions, suggest that Vangl-RhoA-mediated modulation of the cytoskeleton in leader cells is a significant contributor to the invasive nature and metastatic dissemination of primary tumor cells.

## Conclusions

We conclude that Vangl-dependent Wnt/PCP signaling promotes breast cancer collective cell migration independent of breast tumor subtype, pointing to a central role for this pathway in breast cancer progression. Consistently, Wnt/PCP signaling facilitates distant metastasis but not primary tumor growth in a genetically engineered mouse model of breast cancer. As a developmental pathway that becomes reactivated by tumor cells to promote their malignancy, we propose that therapeutic interference with pathway function could specifically thwart metastatic progression in breast cancer patients with advanced disease.

## Supplementary Information

**Additional file 1. Supplementary Video 1. RhoA-FRET biosensor video of MCF7-Control.** Representative videos of spatial activity profiles of RhoA in collectively migrating MCF7 cells stably expressing RhoA-FRET biosensor for Control over 12 hours. Color bars indicate the range of RhoA-FRET biosensor ratios. Scale bar = 25 μm.

**Additional file 2. Supplementary Video 2. RhoA-FRET biosensor video of MCF7-shVangl2-1.** Representative videos of spatial activity profiles of RhoA in collectively migrating MCF7 cells stably expressing RhoA-FRET biosensor for shVangl2-1 over 12 hours. Color bars indicate the range of RhoA-FRET biosensor ratios. Scale bar = 25 μm.

**Additional file 3. Supplementary Video 3. RhoA-FRET biosensor video of MCF7-shVangl2-2.** Representative videos of spatial activity profiles of RhoA in collectively migrating MCF7 cells stably expressing RhoA-FRET biosensor for shVangl2-2 over 12 hours. Color bars indicate the range of RhoA-FRET biosensor ratios. Scale bar = 25 μm.

**Additional file 4.** Supplementary Figures.

**Additional file 5.** Uncropped Western blots.

## Data Availability

All data generated or analyzed during this study are included in this published article and its supplementary information files.

## Abbreviations

Dvl: Dishevelled
EMT: Epithelial–mesenchymal transition
FRET: Fluorescence resonance energy transfer
Fzd: Frizzled
K14: Cytokeratin-14
Lp: Looptail
Pk: Prickle
TNBC: Triple-negative breast cancer
Wnt/PCP: Wnt/Planar cell polarity
