# Supplementary material for: Vangl-dependent Wnt/planar cell polarity signaling mediates collective breast carcinoma motility and distant metastasis
Source: Breast Cancer Res. 2023 May 5;25:52. doi: 10.1186/s13058-023-01651-2 (PMC10163820; doi:10.1186/s13058-023-01651-2)
Supplement: Supplementary file 4 — Additional file 4. Supplementary Figures. [file 13058_2023_1651_MOESM4_ESM.pdf]

Supplementary Figure 1

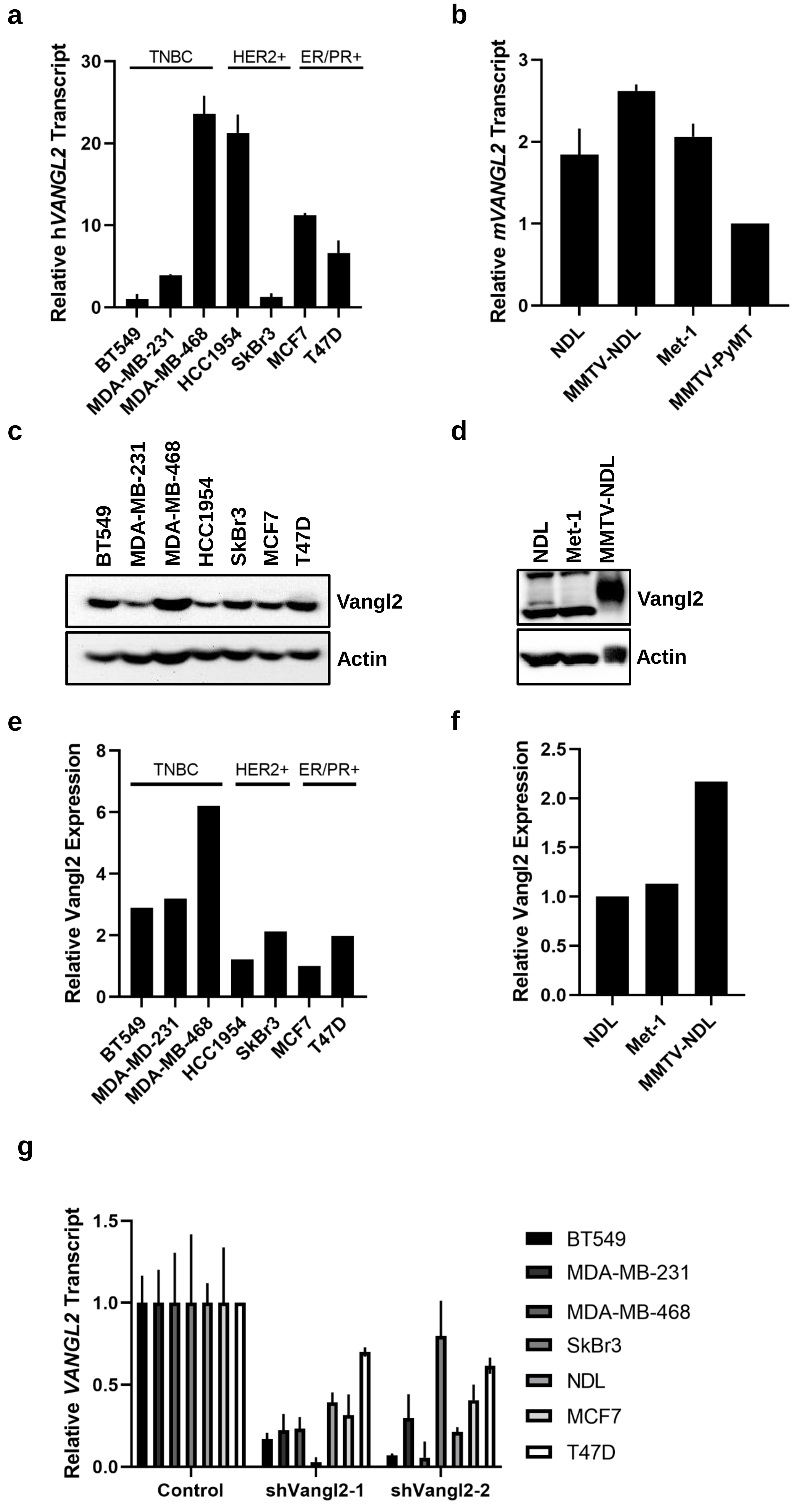

Supplementary Figure 1. Relative Vangl2 Transcript and Protein in human and mouse cell lines and shVangl2 cell lines. **a** Relative *VANGL2* transcript in diverse human breast cancer cell lines. **b** Relative *hVangl2* transcript in *MMTV-NDL* tumors and NDL cell line derived from this model and *MMTV-PyMT* tumors and Met-1 cell line derived from this model. **c-d** Representative Western blots of Vangl2 protein in diverse human **(c)** and mouse **(d)** cell lines and tissues. **e-f** Quantification of human **(e)** and mouse **(f)** Vangl2 protein abundance. **g** Relative *VANGL2* transcript in BT549, MDA-MB-468, MDA-MB-231, SkBr3, NDL, MCF7, and T47D cells stably expressing Control, shVangl2-1, or shVangl2-2 by *q*-PCR.

Supplementary Figure 2

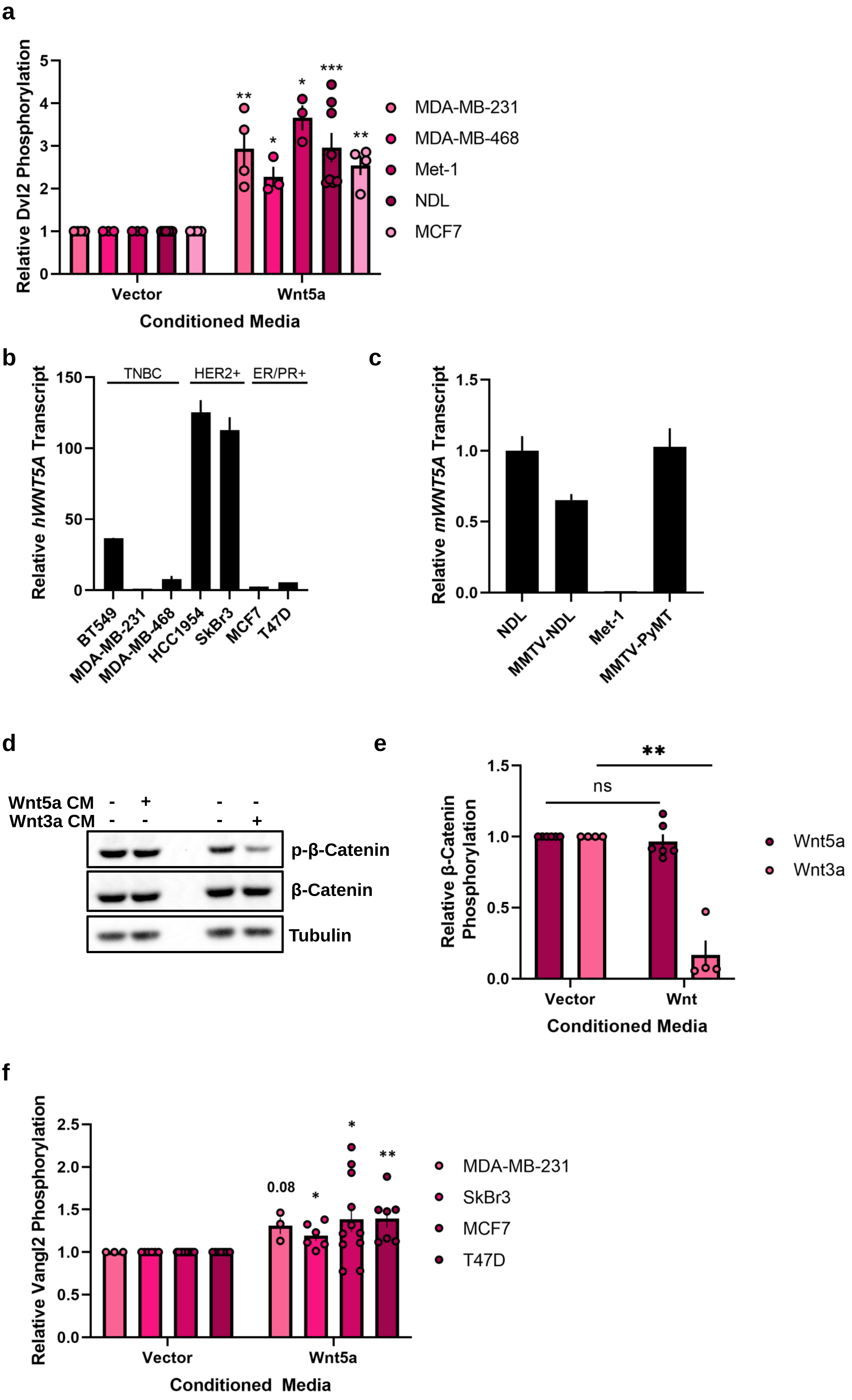

Supplementary Figure 2. Supporting materials for Figure 1. a Quantification of relative Dvl2 phosphorylation in MDA-MB-231, MDA-MB-468, Met-1, NDL, and MCF7 cells stimulated with Vector- or Wnt5a-conditioned media for 1 hour (MDA-MB-231 n=4, p=0.0199, MDA-MB-468 n=3, p=0.0327, Met-1 n=3, p=0.0121, NDL n=8, p=0.0007, MCF7 n=4, p=0.0067) **b** Relative *WNT5A* transcript in diverse human breast cancer cell lines. **c** Relative *hWnt5a* transcript in *MMTV-NDL* tumors and NDL cell line derived from this model and *MMTV-PyMT* tumors and Met-1 cell line derived from this model. **d-e** MCF7 cells stimulated with Vector- or Wnt5a-conditioned media or Vector- or Wnt3a-conditioned media for 1 hour blotted for β-Catenin and phospho-β-Catenin (Ser33/37/Thr41) **(d)** and quantification of relative phosho-β-Catenin (Ser33/37/Thr41) (control- vs Wnt5a-conditioned media n=6, p=0.5419, control- vs Wnt3a-conditioned media n=4, p=0.0038) **(e)**. **f** Quantification of relative Vangl2 phosphorylation in MDA-MB-231, SkBr3, T47D, and MCF7 cells stimulated with Vector- or Wnt5a-conditoned media for 1 hour (MDA-MB-231 n=4, p=0.0846, SkBr3 n=6, p=0.0220, T47D n=7, p=0.0097, and MCF7 n=9, p=0.0246). Bar graphs represent the mean ± sem of experimental replicates (n). Significance was determined by a two-sided unpaired t-test with Welch's correction, *$p < 0.05$, **$p < 0.01$, ***$p < 0.001$, ****$p < 0.0001$.

Supplementary Figure 3

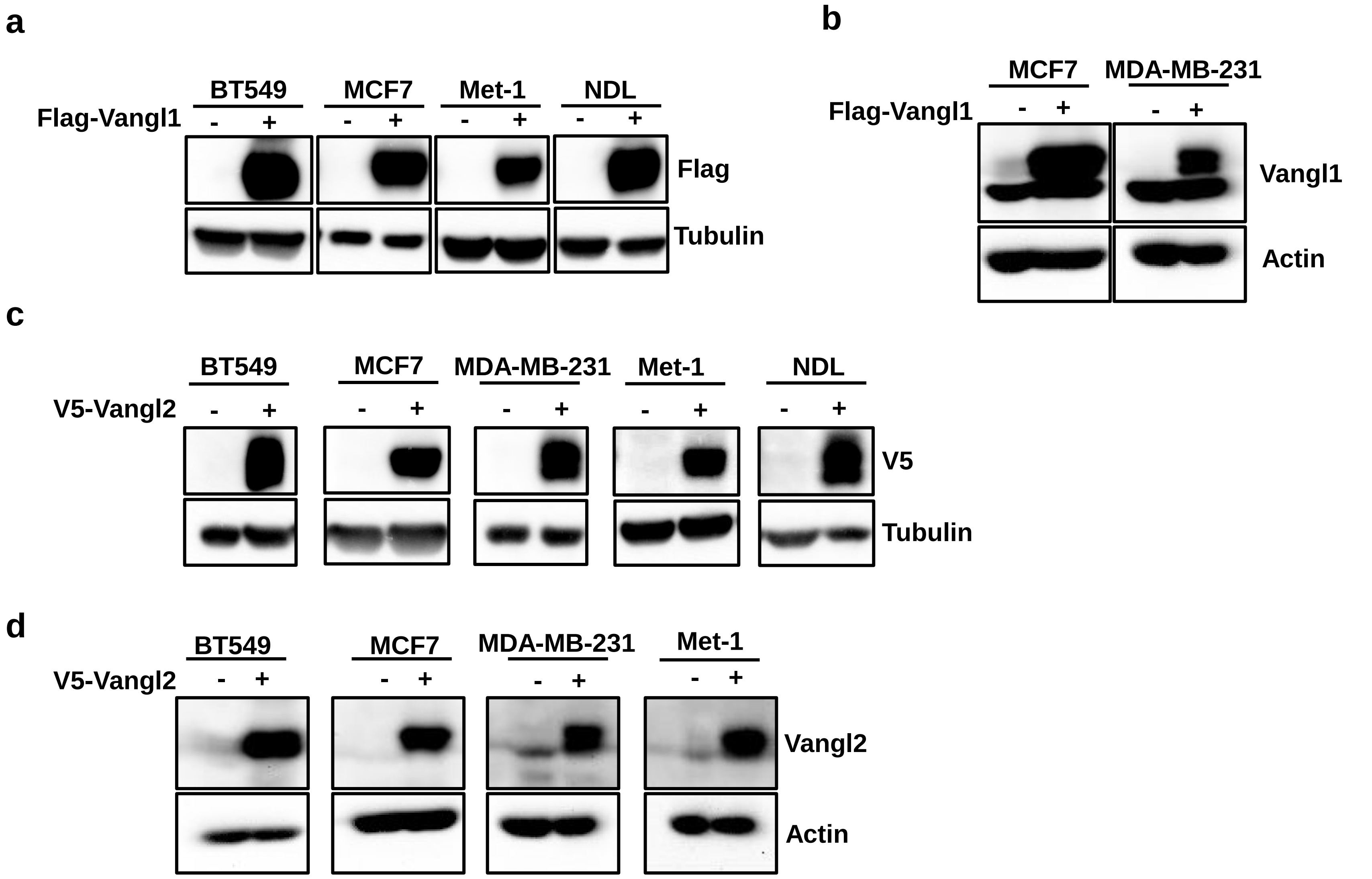

Supplementary Figure 3. Overexpression of Wnt/PCP components. a-d Breast cancer cell lines stably overexpressing Flag-Vangl1 blotted for Flag **(a)** and Vangl1 **(b)** or V5-Vangl2 blotted for V5 **(c)** and Vangl2 **(d).**

Supplementary Figure 4

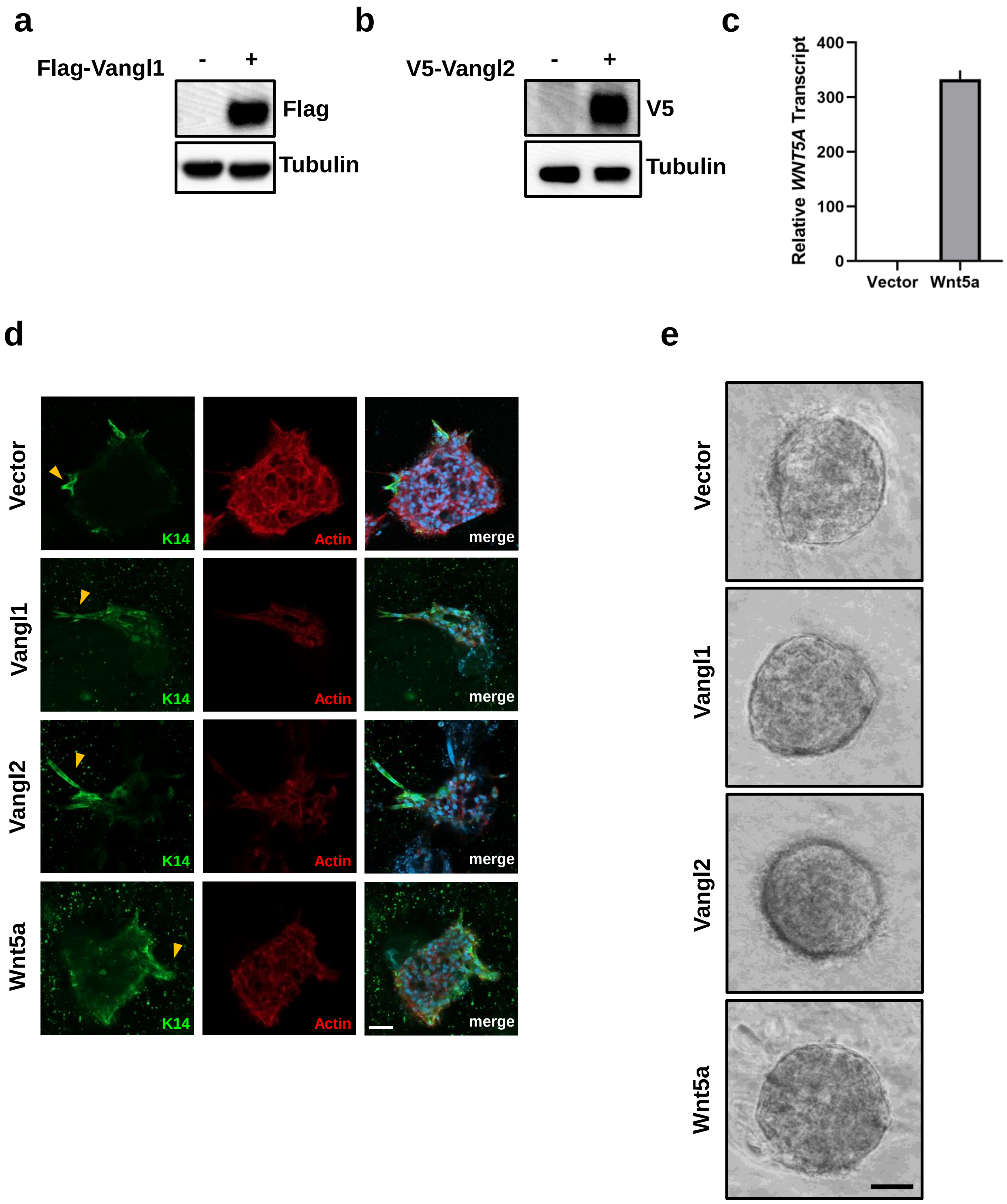

Supplementary Figure 4. Supporting materials for Figure 3. a-c *MMTV-PyMT*-derived tumor organoid cells stably overexpressing Vangl1, Vangl2, or Wnt5a were assessed for Flag-Vangl1 **(a)** or V5-Vangl2 **(b)** expression by Western blot or *WNT5A* transcript by qPCR **(c)**. **d** Representative confocal images of Vector-, Vangl1-, Vangl2-, or Wnt5a-expressing PyMT-derived organoids stained with K14: green, Actin: red, and DAPI: blue. Yellow arrows: K14+ collectively invading protrusion. **e** Representative images of Vector-, Vangl1-, Vangl2-, and Wnt5a-expressing *MMTV-PyMT*-derived tumor organoids in collagen in the absence of *b*FGF, scale bar=50μm.

Supplementary Figure 5

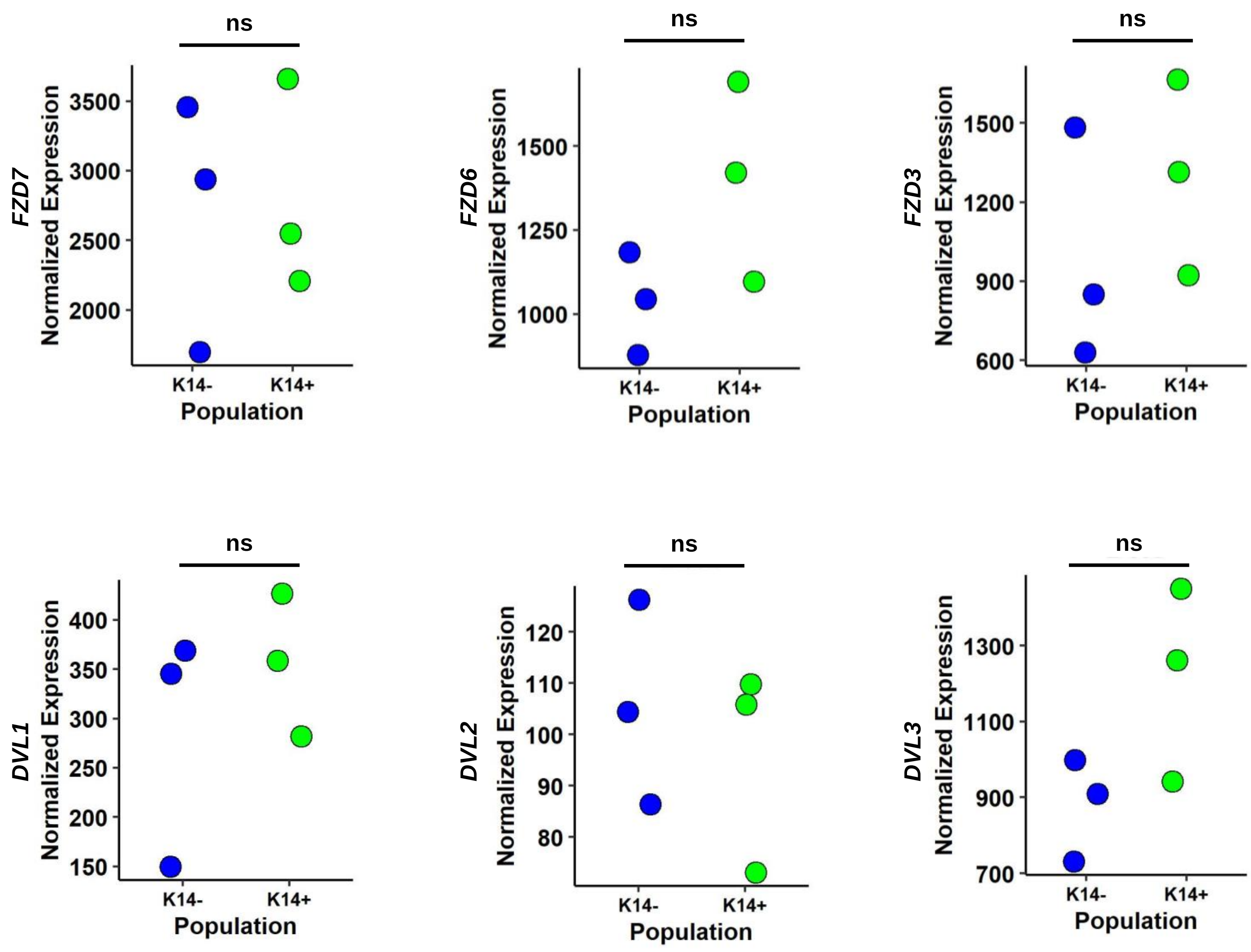

Supplementary Figure 5. Analysis of RNA-sequencing data set SRP066316 from NCBI Sequence Read Archive. *Fzd7*, *Fzd6, Fzd3, Dvl1, Dvl2*, and *Dvl3* transcript in K14-negative and K14-postive cells derived from *MMTV-PyMT* tumors.

Supplementary Figure 6

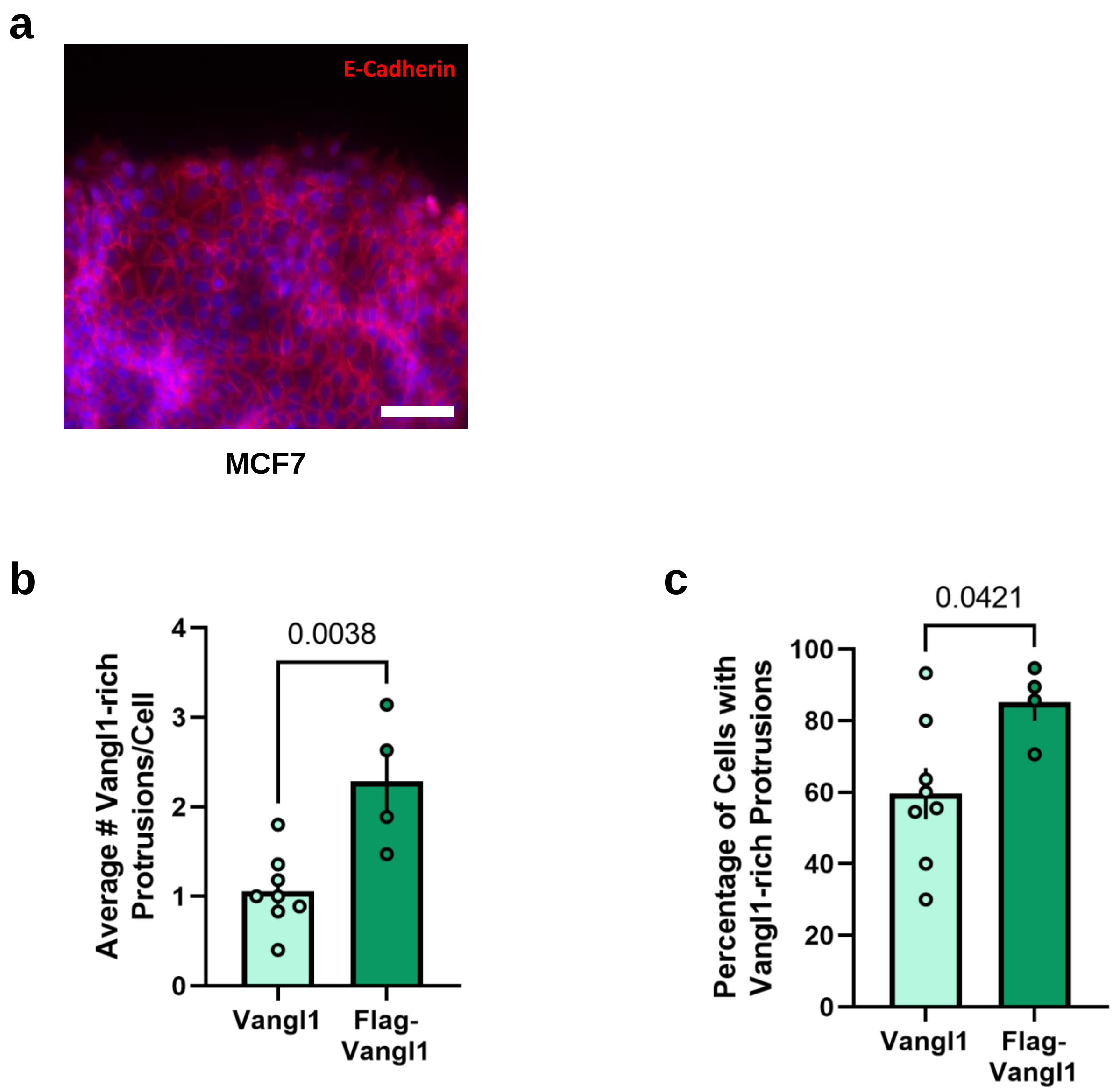

Supplementary Figure 6. Supporting materials for Figure 4. a Representative confocal images of collectively migrating MCF7 cells stained for E-Cadherin: orange and DAPI: blue, scale bar = 50μm. **b** Quantification of the average number of Vangl1-rich protrusions per leading-edge cell in collectively migrating MCF7-Vector and MCF7-Flag-Vangl1 cells (MCF7-Vector *n*=8 *scratches quantified,* MCF7-Flag-Vangl1 *n*=4 *scratches quantified*, *p*=0.0038) **c** Quantification of the percentage of leading-edge cells with Vangl1-rich protrusions (MCF7-Vector *n*=8, MCF7-Flag-Vangl1 *n*=4, *p*=0.0421). Bar graphs represent the mean ± sem of experimental replicates (*n*). Significance was determined by a two-sided unpaired *t*-test with Welch's correction.

Supplementary Figure 7

a

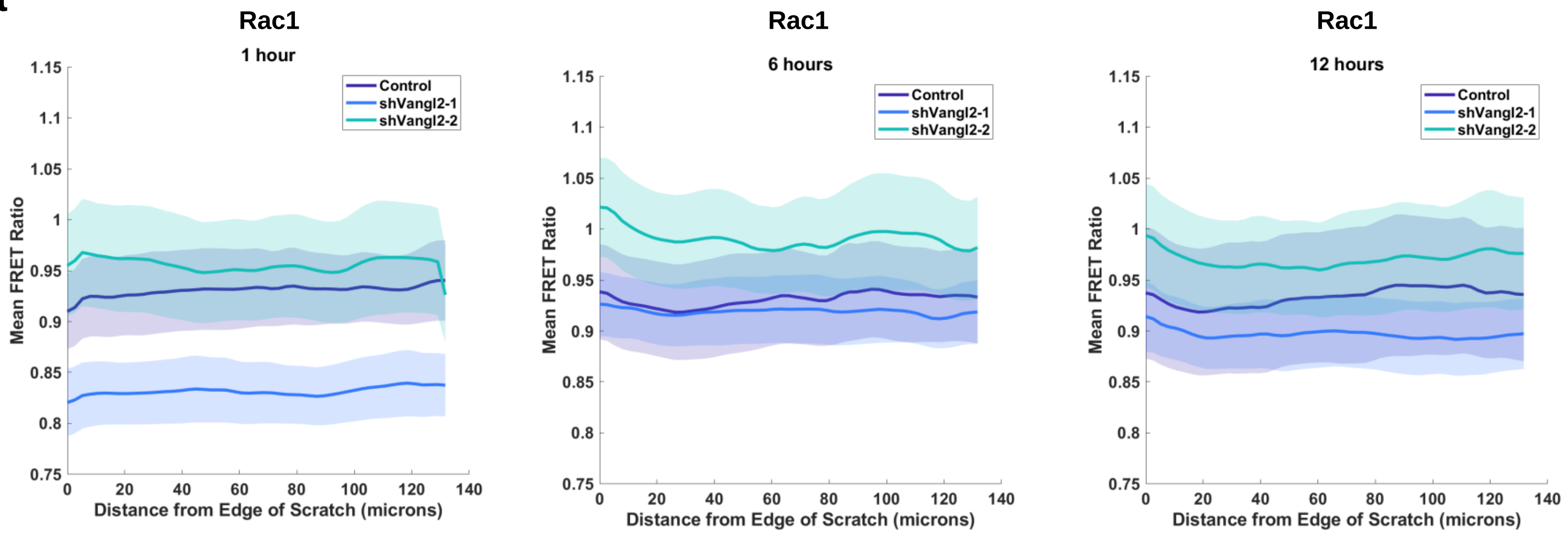

b

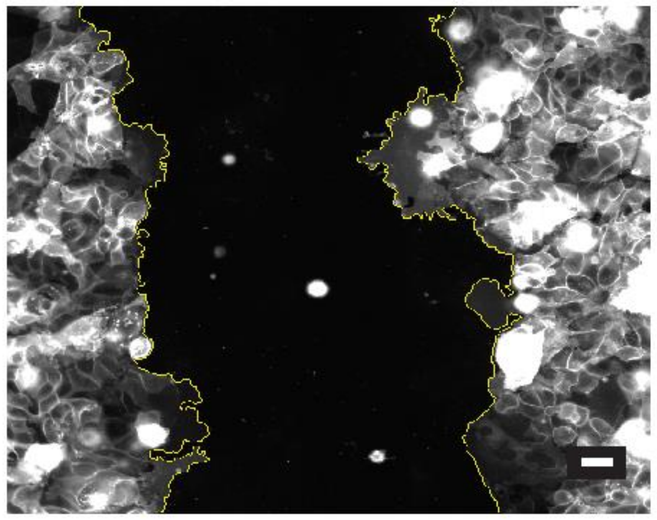

c

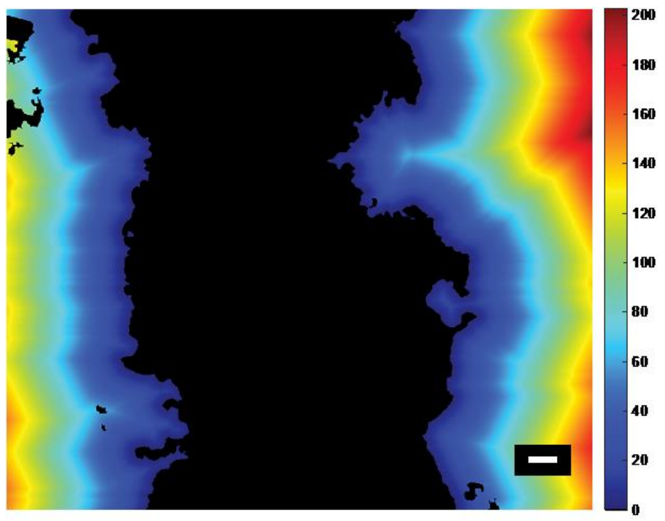

d

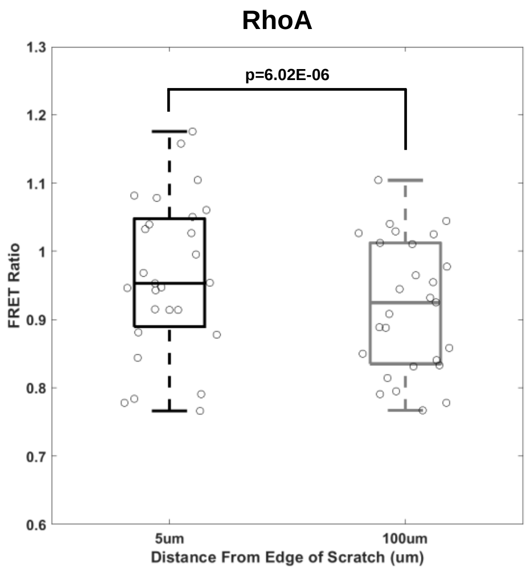

Supplementary Figure 7. Supporting materials for Figure 5. a Rac1 activity as a function of distance in microns from the leading-edge of collectively migrating MCF7 cells stably expressing Vector (*n*=27 wells), shVangl2-1 (*n*=24 wells), or shVangl2-2 (*n*=25 wells) at 1, 6, and 12 hours of migration, error bars indicate ± sem. **b-c** Representative example of custom MATLAB script identifying the leading-edge of a migrating cohort of MCF7 cells **(b)** and binned migrating cells based on their distance from the edge of the scratch in microns, where color bar indicates the distance from the leading-edge of the scratch **(c)**, scale bars=25μm. **d** RhoA activity in MCF7 cells stably expressing the RhoA biosensor and Control at 5μm and 100μm from the edge the of scratch (*n*=27, *p*=6.02E-06), significance was determined by a two-sided paired *t*-test.

Supplementary Figure 8

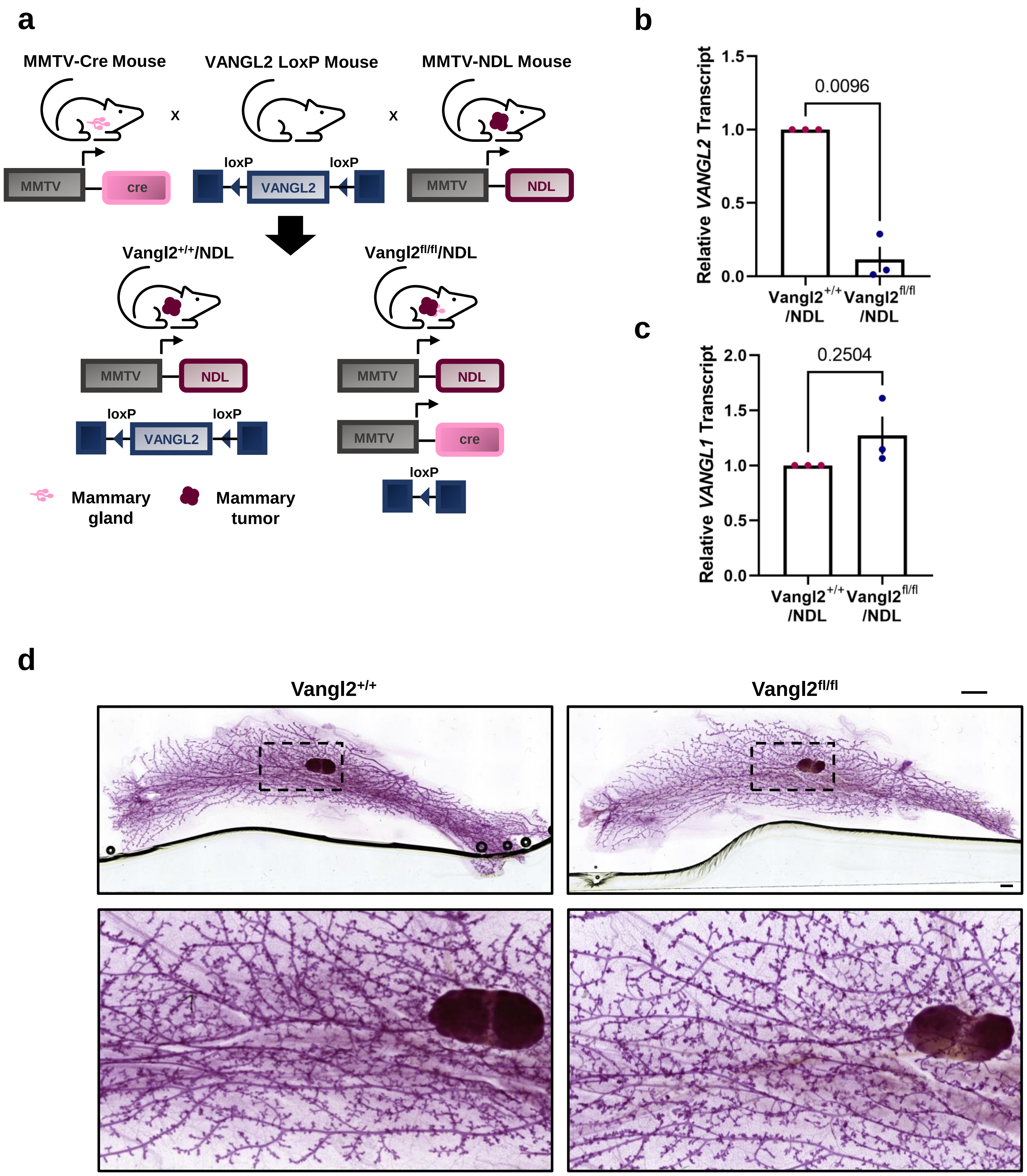

Supplementary Figure 8. Supporting materials for Figure 6. a Summary of the transgenic mouse strategy employed to assess Vangl2 deletion in mammary tumorigenesis. **b-c** *VANGL2* **(b)** and *VANGL1* **(c)** transcript from Vangl2$^{fl/fl}$/NDL and Vangl2$^{+/+}$/NDL primary tumors by *q*PCR from three tumors of independent biological sources per genotype. **d** Carmine alum stained mammary whole mounts from estrus matched 20-week-old Vangl2$^{+/+}$ and Vangl2$^{fl/fl}$ mice, demonstrating no detectable differences in gland architecture in adult virgin mice. Scale bars = 1mm (top panels) or 500µm (bottom panels).

Supplementary Figure 9

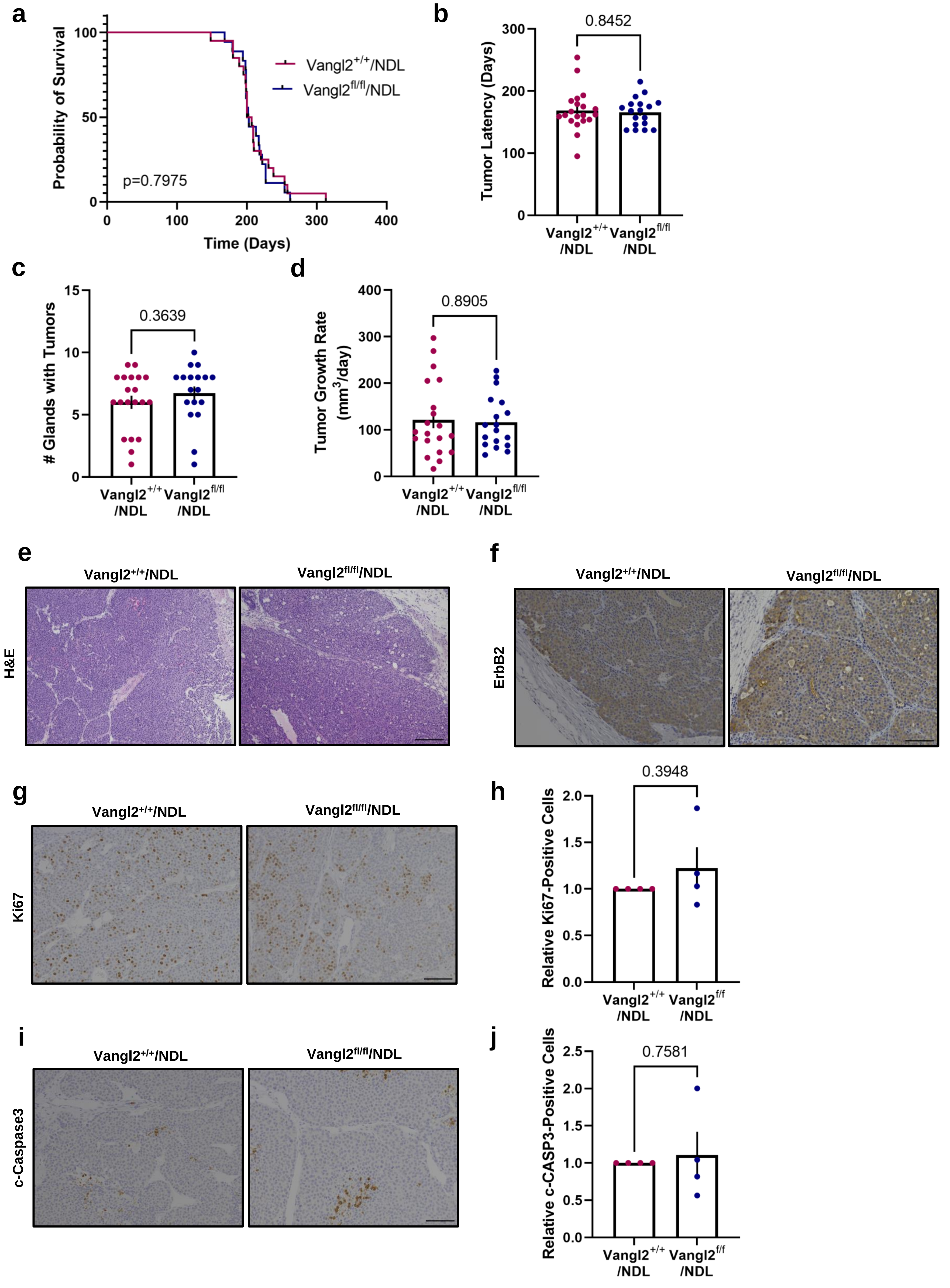

Supplementary Figure 9. Vangl2 deletion does not impact primary tumor growth characteristics or histology. a-d Survival curves and bar graphs depicting Vangl2/NDL tumor initiation and growth characteristics. Probability of survival **(a),** tumor latency **(b),** number of glands with tumors **(c),** and tumor growth rate **(d)** for Vangl2$^{+/+}$/NDL (*n*=20) and Vangl2$^{fl/fl}$/NDL (*n*=20) tumor-bearing animals. **e-f** Representative images are depicted of formalin fixed, paraffin embedded sections from Vangl2$^{+/+}$/NDL and Vangl2$^{fl/fl}$/NDL primary tumors stained with H&E **(e)** or following immunodetection with ErbB2 **(f)**, scale bar=200µm. **g-j** Representative images of Vangl2$^{+/+}$/NDL and Vangl2$^{fl/fl}$/NDL primary tumor tissues following immunodetection of proliferation marker Ki67 **(g)** with quantification of Ki67-positive cells (*n*=4) **(h)** and apoptosis marker cleaved caspase-3 **(i)** with quantification of c-Caspase 3-positive cells (*n*=4) **(j)**, scale bar=100µm. Significance determined by Log-rank **(a)** or Mann-Whitney test **(c-d)** or two-sided unpaired *t*-test with Welch's correction **(h,j)**. All bar graphs represent the mean ± sem of experimental replicates (*n*).

Supplementary Figure 10

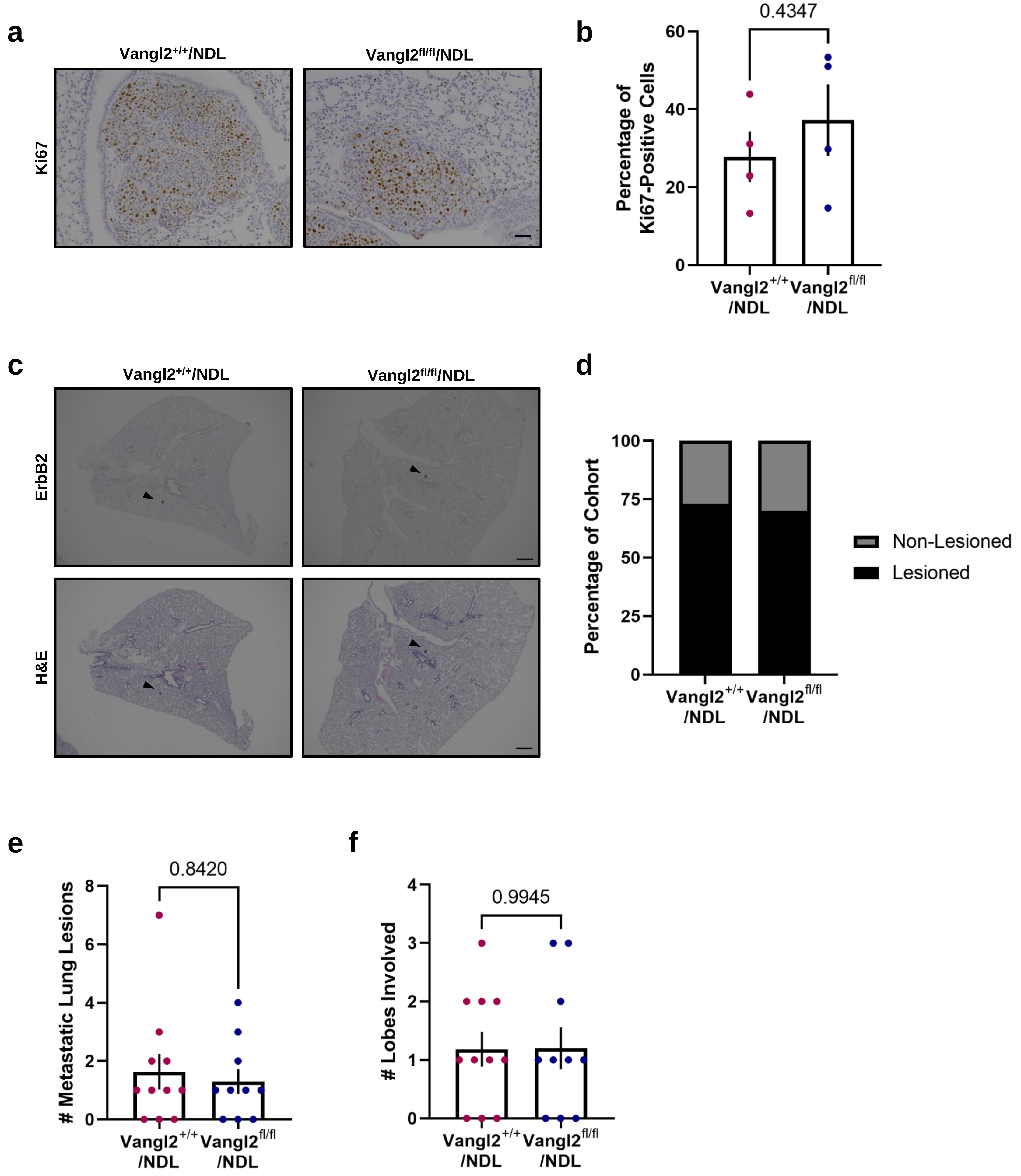

Supplementary Figure 10. Vangl2 is dispensable for metastatic lesion colonization and proliferation. a-b Metastatic lung lesions of Vangl2$^{+/+}$/NDL (*n*=4) and Vangl2$^{fl/fl}$/NDL tumor-bearing mice (*n*=4) were evaluated for proliferative capacity by immunodetection of Ki67 **(a)** and the percentage of Ki67-positive cells was quantified **(b). c** Representative images of formalin fixed, paraffin embedded sections of lungs from FvB/NJ mice receiving Vangl2$^{+/+}$/NDL or Vangl2$^{fl/fl}$/NDL cells via the tail vein following immunodetection of ErbB2 (top panel) and H&E staining (bottom panel). Examples of ErbB2-positive metastatic lung lesions are denoted by black arrowheads, scale bar =500μm. **d-f** Lung lobes (5 lobes per mouse) from FvB/NJ mice receiving Vangl2$^{+/+}$/NDL or Vangl2$^{fl/fl}$/NDL cells via the tail vein were evaluated by histology for the occurrence of metastatic lesions for Vangl2$^{+/+}$/NDL (*n*=11) and Vangl2$^{fl/fl}$/NDL (*n*=10) cohorts. The number of mice bearing metastatic lesions **(d)**, numbers of metastatic lesions **(e)**, and numbers of lung lobes involved **(f)** were assessed. Significance was determined by Mann-Whitney test and bar graphs represent the mean ± sem of experimental replicates (*n*).

Supplementary Movies 1-3

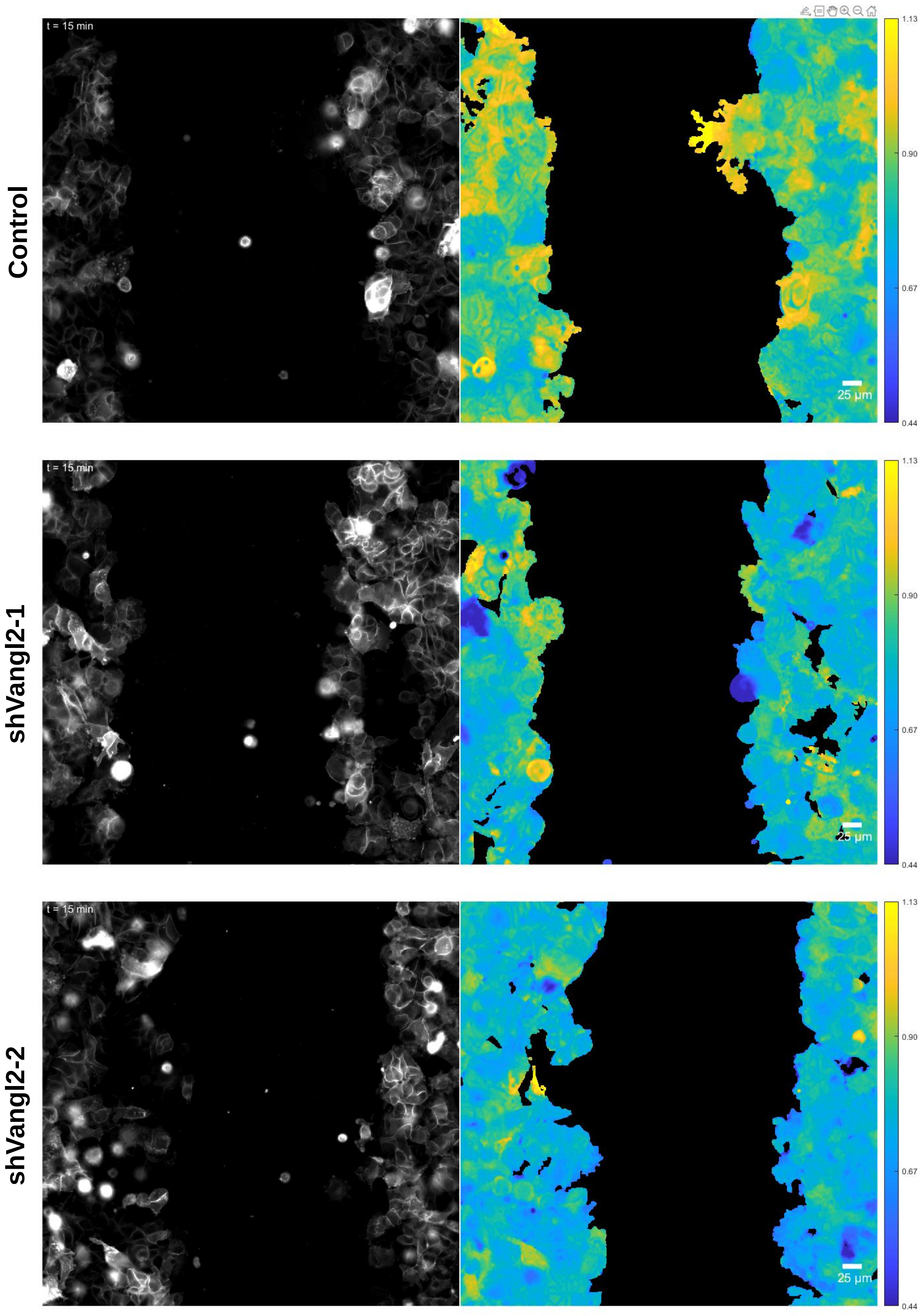

Supplementary Video 1. RhoA-FRET biosensor video of MCF7-Control. Representative videos of spatial activity profiles of RhoA in collectively migrating MCF7 cells stably expressing RhoA-FRET biosensor for Control over 12 hours. Color bars indicate the range of RhoA-FRET biosensor ratios. Scale bar=25μm.

Supplementary Video 2. RhoA-FRET biosensor video of MCF7-shVangl2-1. Representative videos of spatial activity profiles of RhoA in collectively migrating MCF7 cells stably expressing RhoA-FRET biosensor for shVangl2-1 over 12 hours. Color bars indicate the range of RhoA-FRET biosensor ratios. Scale bar=25μm.

Supplementary Video 3. RhoA-FRET biosensor video of MCF7-shVangl2-2. Representative videos of spatial activity profiles of RhoA in collectively migrating MCF7 cells stably expressing RhoA-FRET biosensor for shVangl2-2 over 12 hours. Color bars indicate the range of RhoA-FRET biosensor ratios. Scale bar=25μm.
