# Supplementary material for: Vangl-dependent Wnt/planar cell polarity signaling mediates collective breast carcinoma motility and distant metastasis
Source: Breast Cancer Res. 2023 May 5;25:52. doi: 10.1186/s13058-023-01651-2 (PMC10163820; doi:10.1186/s13058-023-01651-2)
Supplement: Supplementary file 5 — Additional file 5. Uncropped Western blots. [file 13058_2023_1651_MOESM5_ESM.pdf]

**Western Blot Raw Data**

Blots for Figure 1D with relevant bands boxed.

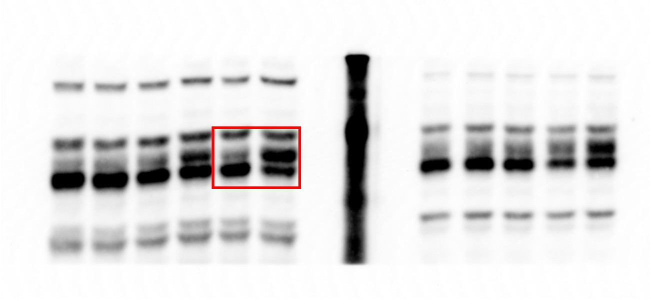

Dvl2, MCF7

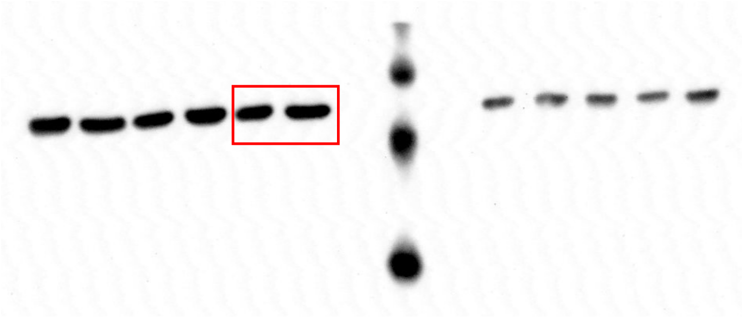

Tubulin, MCF7

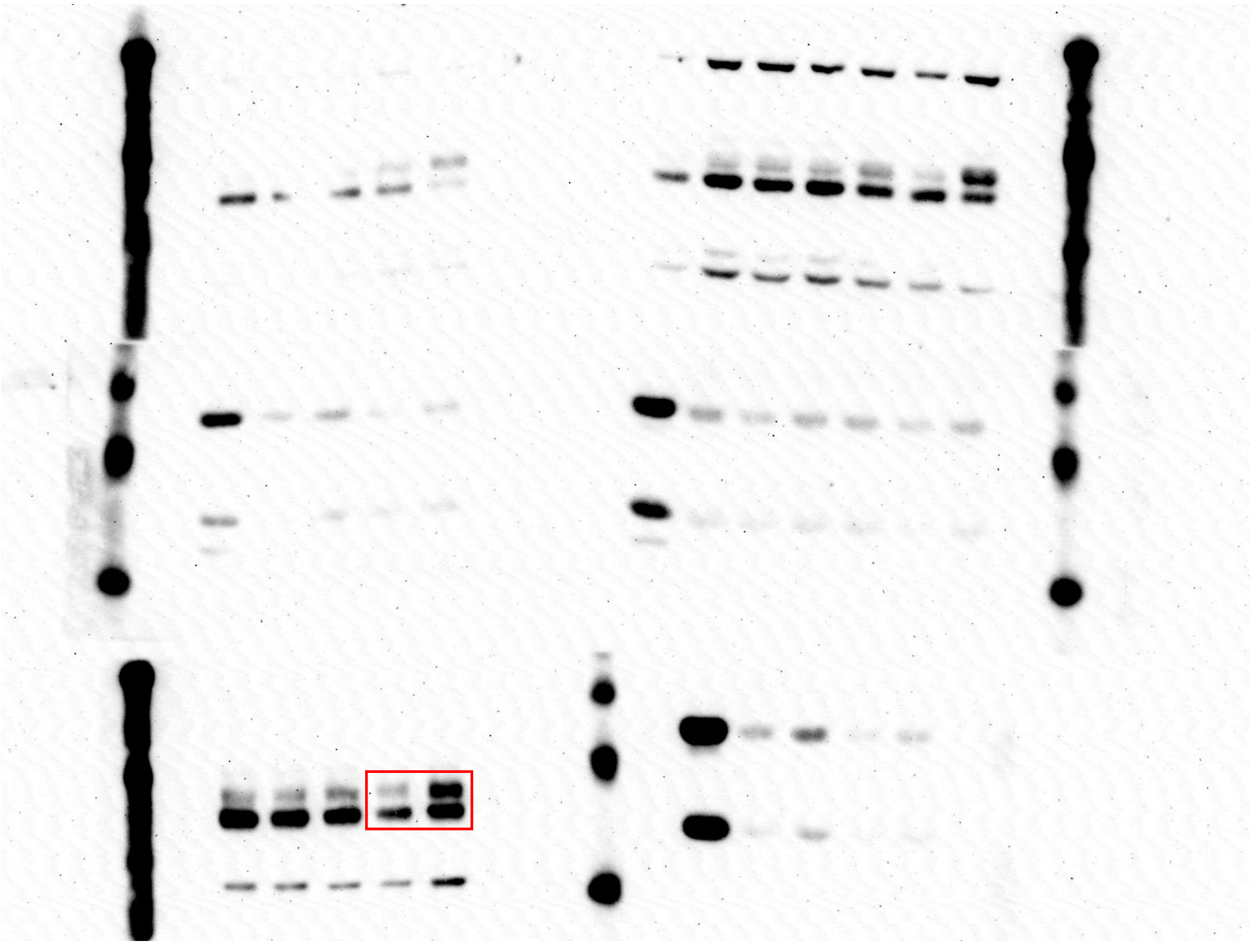

Dvl2, MDA-MB-468

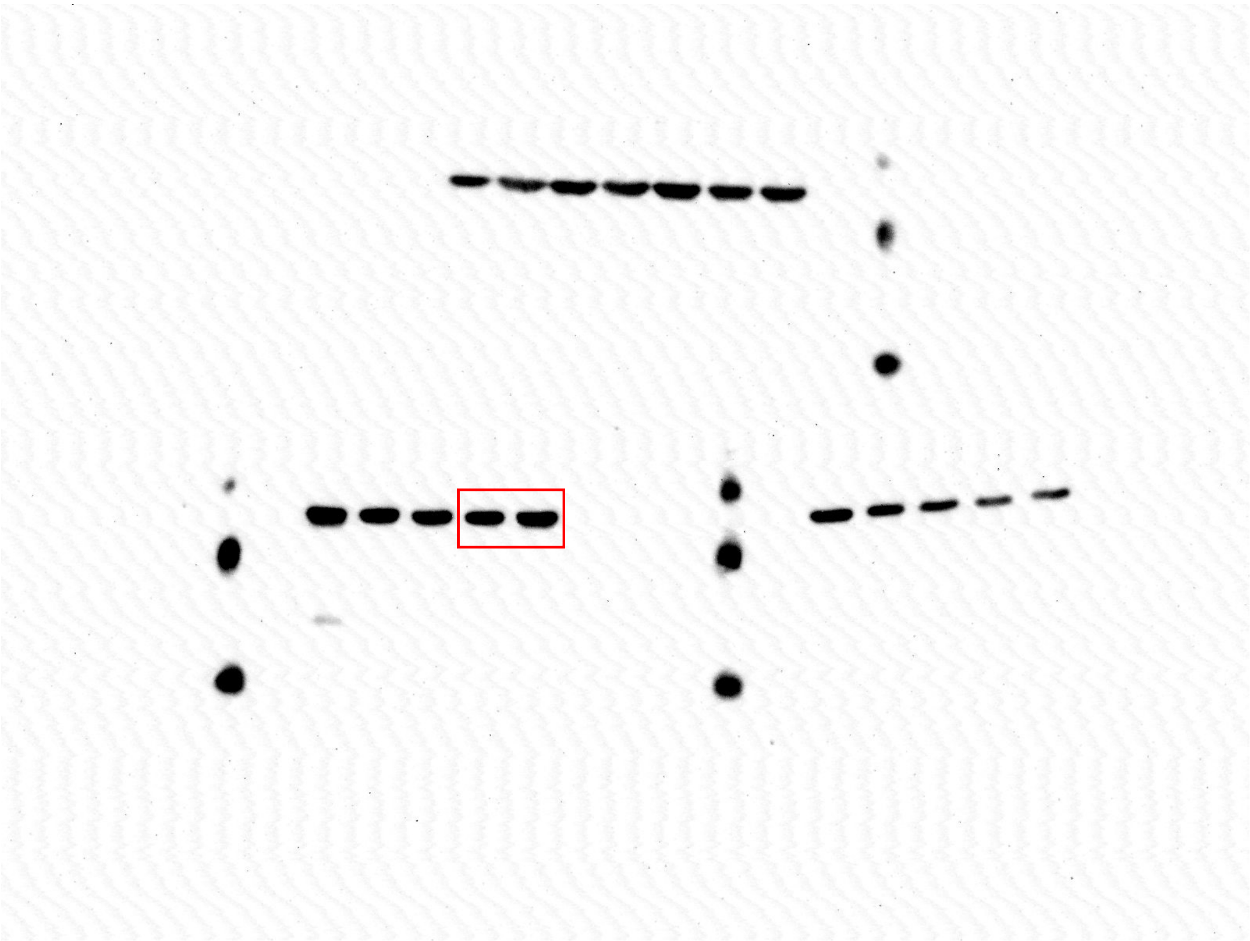

Tubulin, MDA-MB-468

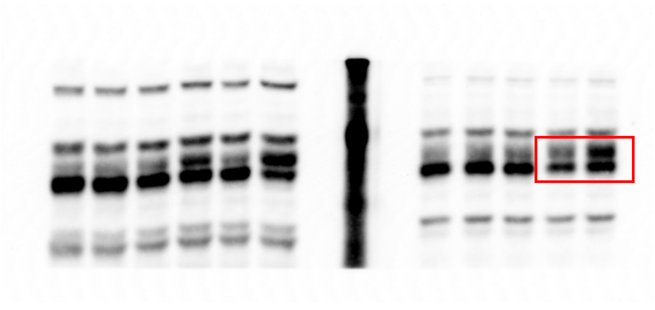

Dvl2, NDL

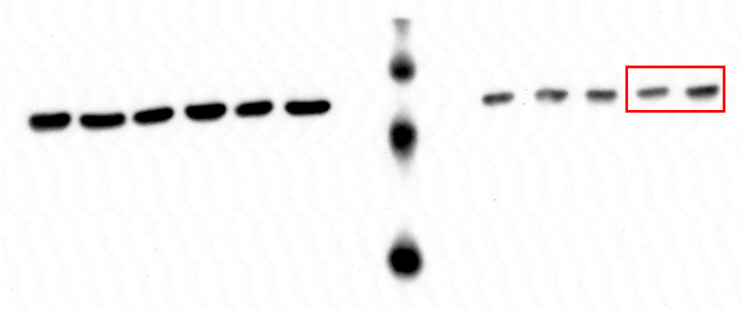

Tubulin, NDL

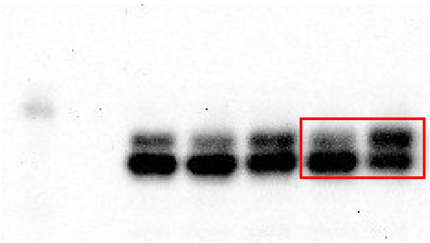
Dvl2, MDA-MB-231

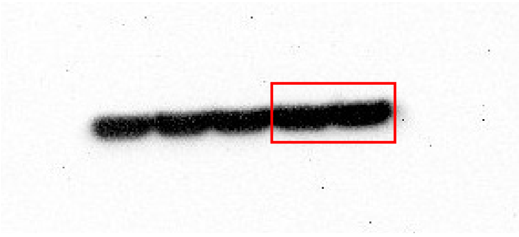
Tubulin, MDA-MB-231

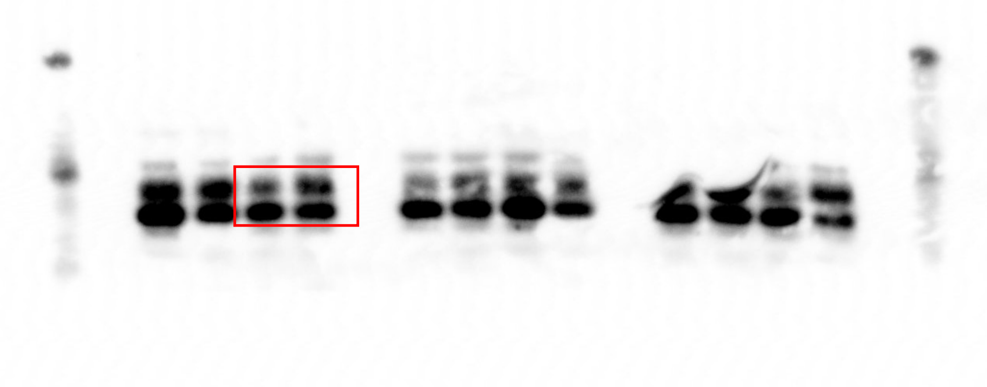
Dvl2, Met-1

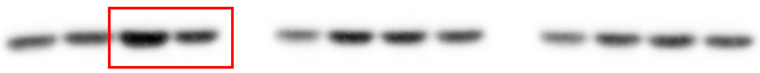
Tubulin, Met-1

Blots for Figure 2E with relevant bands boxed.

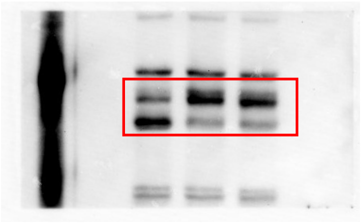

Dvl2

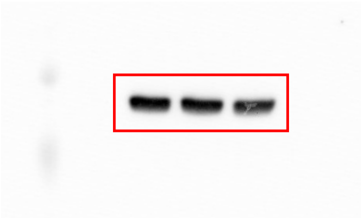

Tubulin

Blots for Figure 2G with relevant bands labeled.

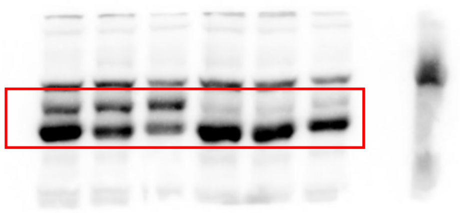

Dvl2

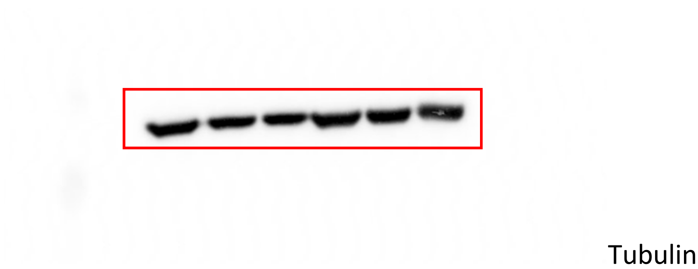

Tubulin

Blots for Supp Figure S1C.

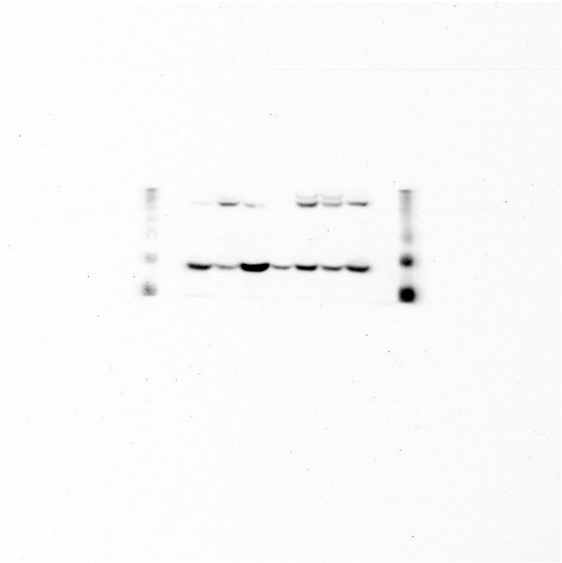

Vangl2

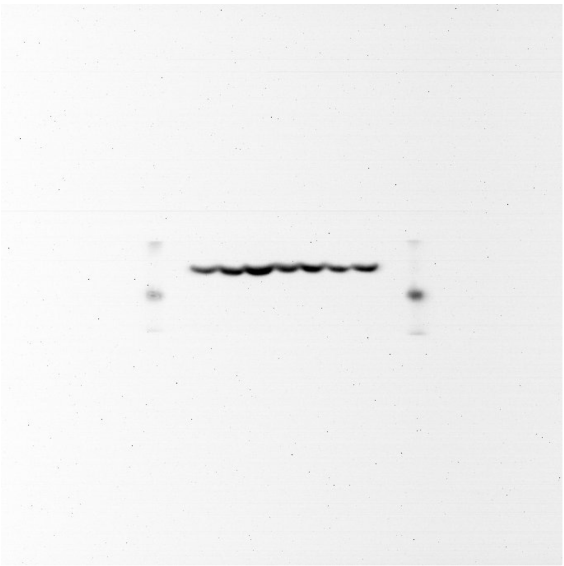

Actin

Blots for Supp Figure S1D.

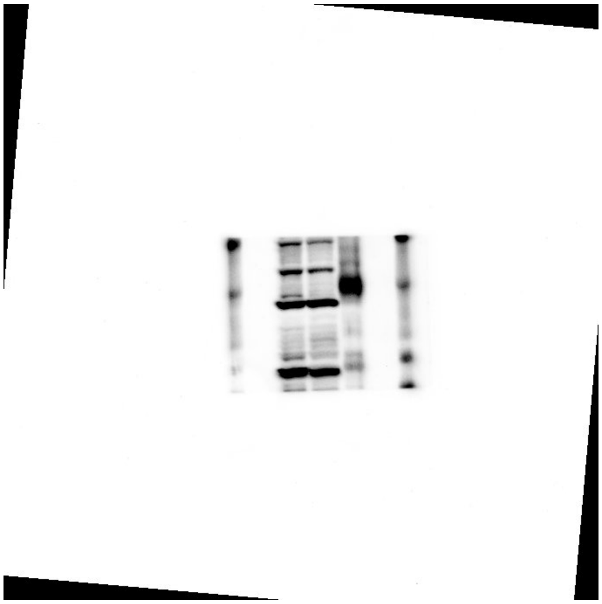

Vangl2

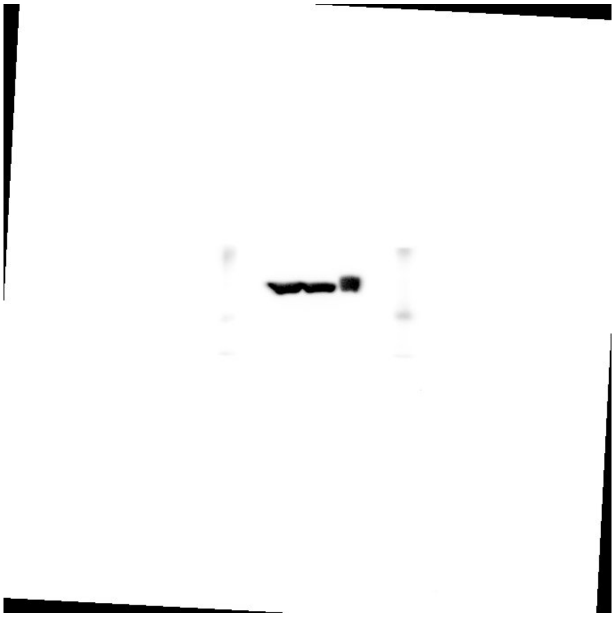

Actin

Blots for Suppl Figure S2D with relevant bands boxed.

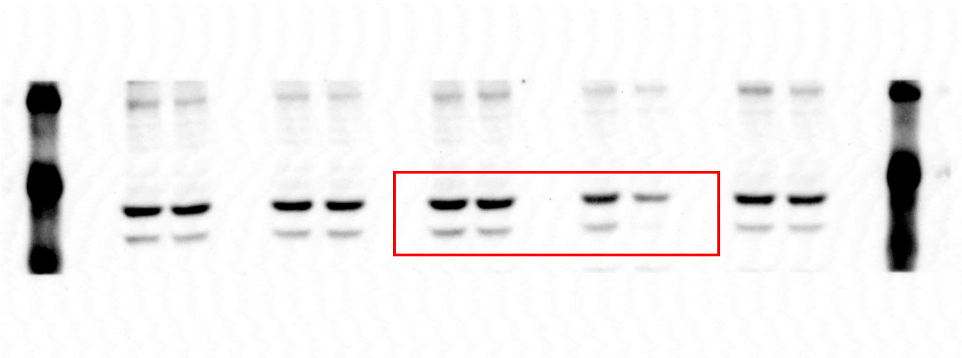

Phospho- β-Catenin

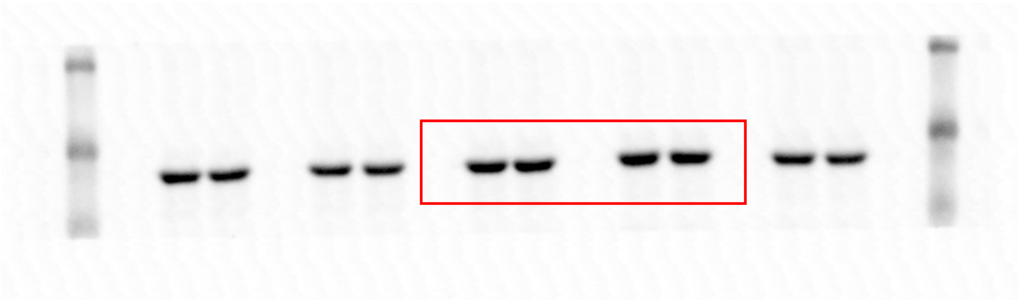

β-Catenin

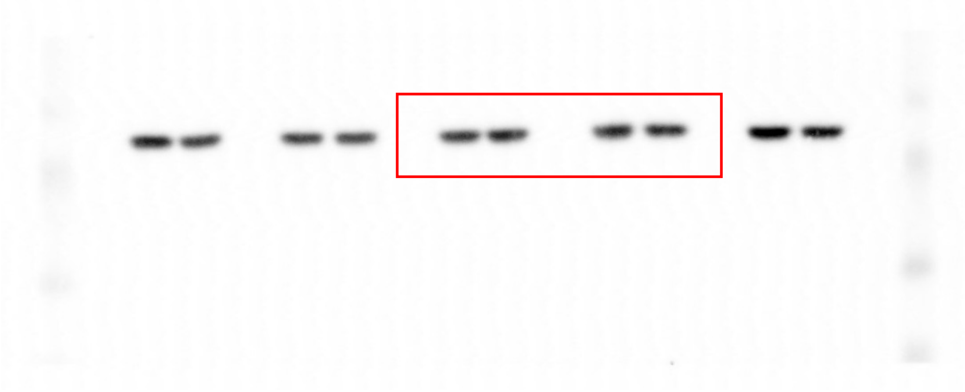

Tubulin

Blots for Supp Figure S3A with relevant bands boxed.

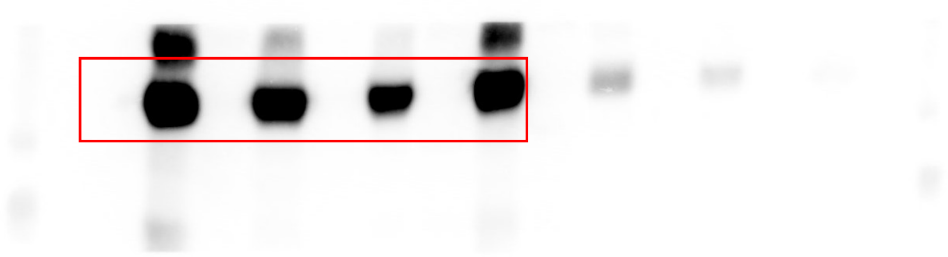

Flag

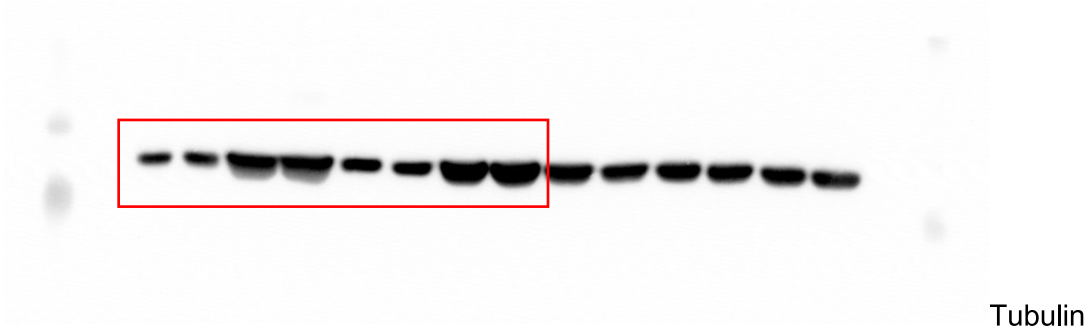

Tubulin

Blots for Supp Figure S3B with relevant bands boxed.

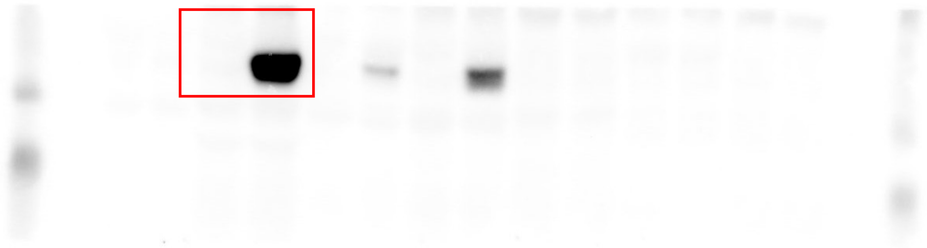

V5, MCF7

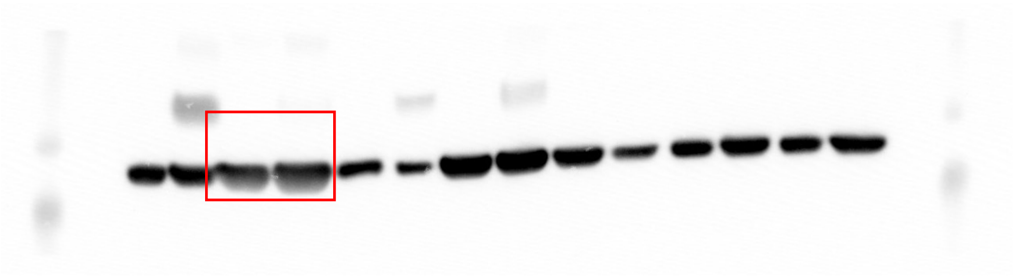

Tubulin, MCF7

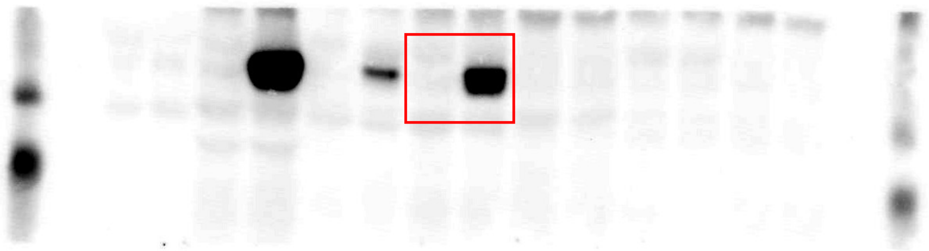

V5, Met-1

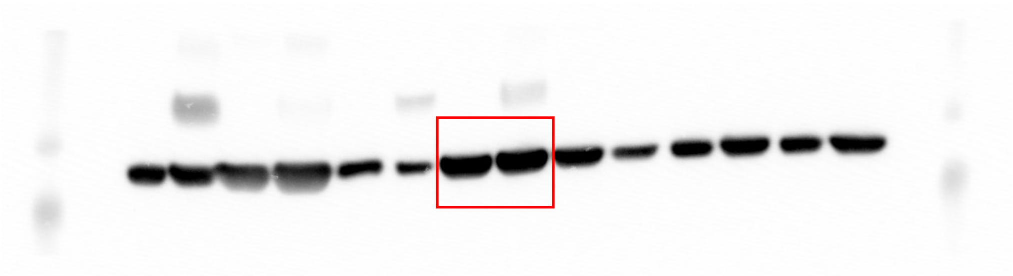

Tubulin, Met-1

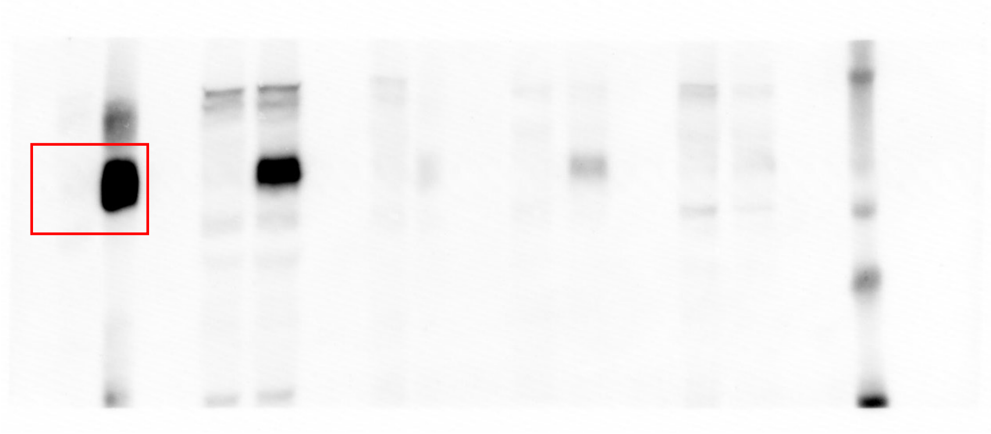

V5, BT549

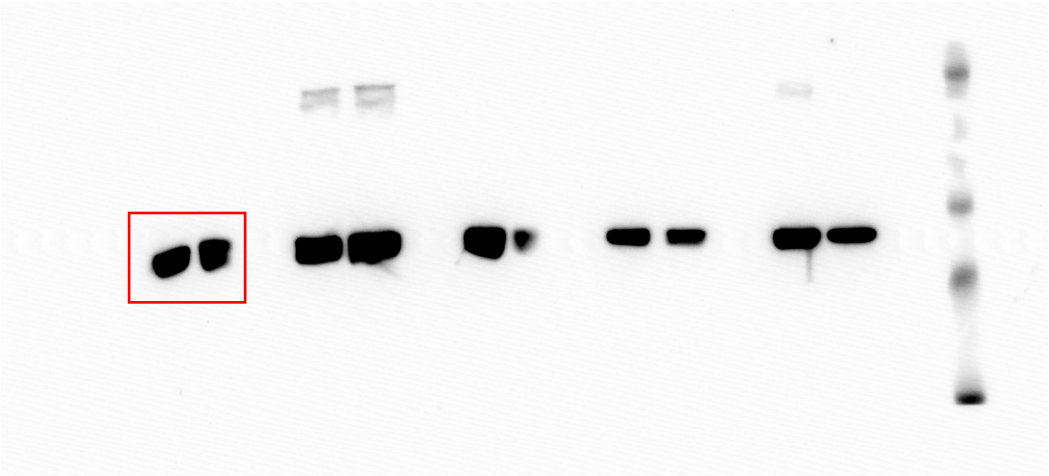

Tubulin, BT549

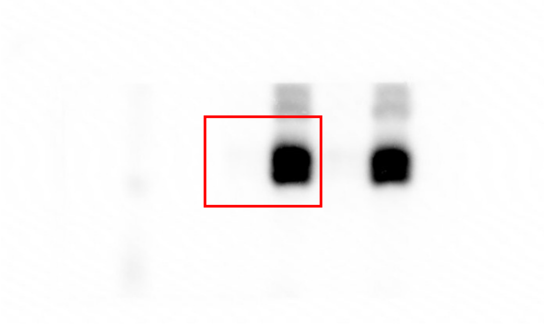

V5, MDA-MB-231

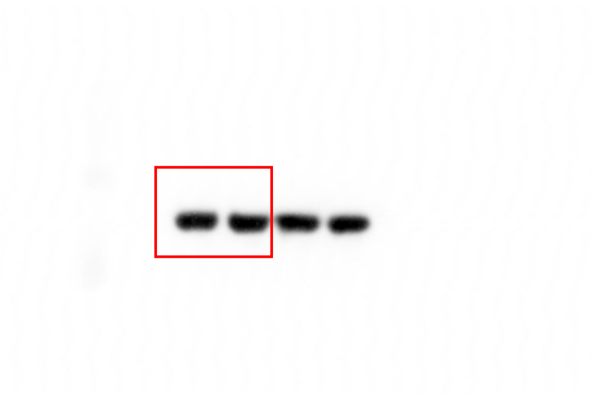

Tubulin, MDA-MB-231

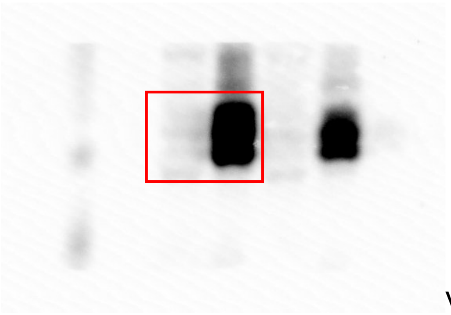

V5, NDL

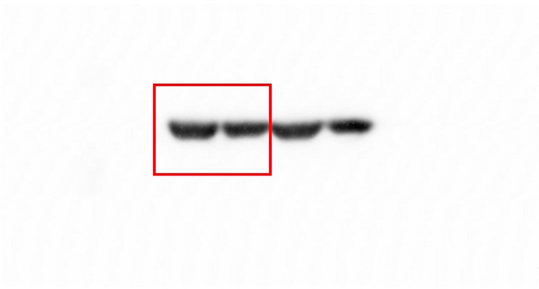

Tubulin, NDL

Blots for Supp Figure S4A with relevant bands boxed.

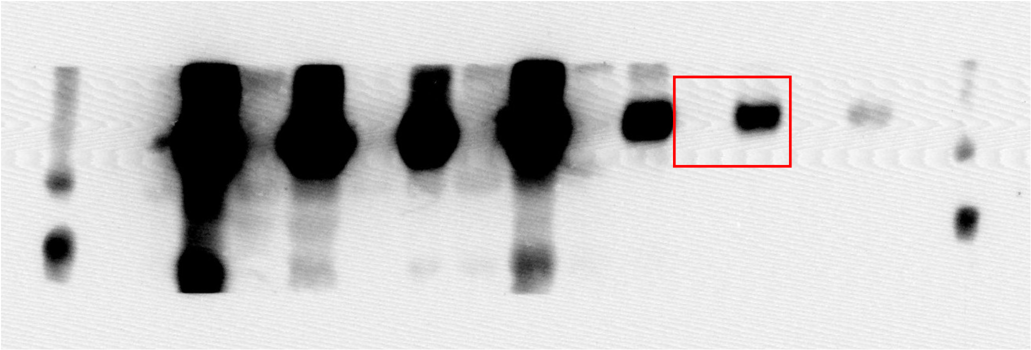

Flag, PyMT

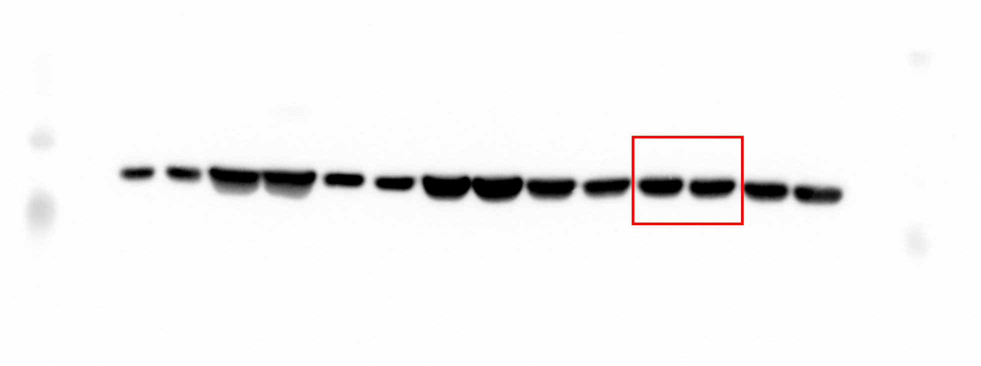

Tubulin, PyMT

Blots for Supp Figure S4B with relevant bands boxed.

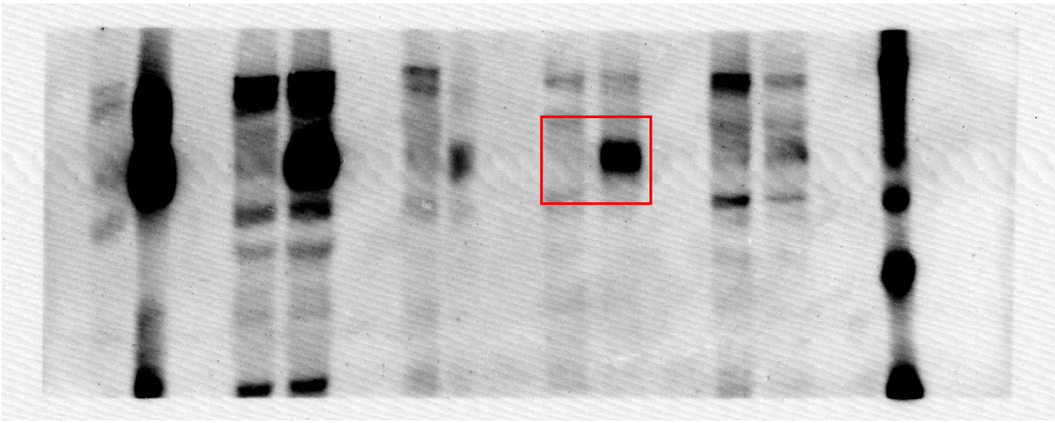

V5, PyMT

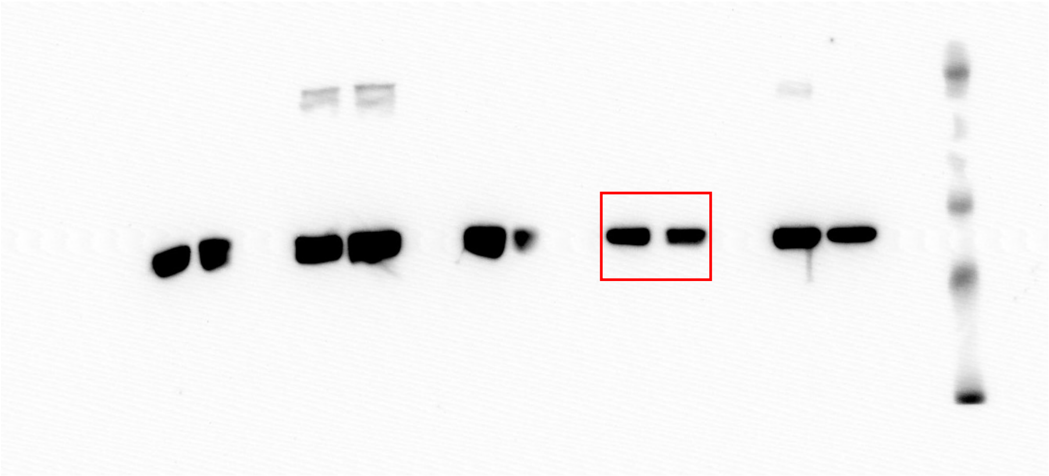

Tubulin, Py
